# An siRNA Screen Identifies the U2 snRNP Spliceosome as a Host Restriction Factor for Recombinant Adeno-associated Viruses

**DOI:** 10.1371/journal.ppat.1005082

**Published:** 2015-08-05

**Authors:** Claire A. Schreiber, Toshie Sakuma, Yoshihiro Izumiya, Sara J. Holditch, Raymond D. Hickey, Robert K. Bressin, Upamanyu Basu, Kazunori Koide, Aravind Asokan, Yasuhiro Ikeda

**Affiliations:** 1 Department of Molecular Medicine, Mayo Clinic, Rochester, Minnesota, United States of America; 2 Department of Dermatology, UC Davis School of Medicine, Sacramento, California, United States of America; 3 Department of Chemistry, University of Pittsburgh, Pittsburgh, Pennsylvania, United States of America; 4 Gene Therapy Center and Department of Genetics, University of North Carolina School of Medicine, Chapel Hill, North Carolina, United States of America; Stony Brook University, UNITED STATES

## Abstract

Adeno-associated viruses (AAV) have evolved to exploit the dynamic reorganization of host cell machinery during co-infection by adenoviruses and other helper viruses. In the absence of helper viruses, host factors such as the proteasome and DNA damage response machinery have been shown to effectively inhibit AAV transduction by restricting processes ranging from nuclear entry to second-strand DNA synthesis. To identify host factors that might affect other key steps in AAV infection, we screened an siRNA library that revealed several candidate genes including the PHD finger-like domain protein 5A (PHF5A), a U2 snRNP-associated protein. Disruption of PHF5A expression selectively enhanced transgene expression from AAV by increasing transcript levels and appears to influence a step after second-strand synthesis in a serotype and cell type-independent manner. Genetic disruption of U2 snRNP and associated proteins, such as SF3B1 and U2AF1, also increased expression from AAV vector, suggesting the critical role of U2 snRNP spliceosome complex in this host-mediated restriction. Notably, adenoviral co-infection and U2 snRNP inhibition appeared to target a common pathway in increasing expression from AAV vectors. Moreover, pharmacological inhibition of U2 snRNP by meayamycin B, a potent SF3B1 inhibitor, substantially enhanced AAV vector transduction of clinically relevant cell types. Further analysis suggested that U2 snRNP proteins suppress AAV vector transgene expression through direct recognition of intact AAV capsids. In summary, we identify U2 snRNP and associated splicing factors, which are known to be affected during adenoviral infection, as novel host restriction factors that effectively limit AAV transgene expression. Concurrently, we postulate that pharmacological/genetic manipulation of components of the spliceosomal machinery might enable more effective gene transfer modalities with recombinant AAV vectors.

## Author Summary

Mammalian cells have developed diverse innate/intrinsic immune strategies to counteract viral infections. Post-entry infection steps of a single-strand DNA virus, adeno-associated virus (AAV), are subject to such restrictions. Here, we screened an siRNA library to identify a novel cellular factor involved in AAV restriction. We found PHF5A, a component of the U2 snRNP mRNA splicing factor, blocks expression from recombinant AAV vectors. Disruption of PHF5A expression specifically enhanced AAV vector performance. Moreover, genetic and pharmacological inhibition of other U2 snRNP proteins, but not spliceosome proteins involved in other splicing steps, strongly increased transgene expression from AAV vectors. Further study demonstrated that U2 snRNP proteins recognize incoming AAV capsids to mediate this cellular restriction at the step after second-strand synthesis. In summary, we identify the U2 snRNP spliceosome complex as novel host factors that effectively restrict recombinant AAV vectors. Considering frequent reorganization of host splicing machinery in DNA virus infections, it is conceivable that U2 snRNP plays a role as a broad spectrum antiviral factor and helper viruses have evolved to counteract this restriction through sequestration of snRNP proteins.

## Introduction

Viral pathogens are known to reorganize different components of the host cell machinery during the course of infection. For instance, adenoviruses have been shown to induce nuclear reorganization of host splicing factors and mislocalization of the DNA damage response machinery [1]. Similarly, herpesviruses can induce sequestration of cellular chaperone proteins and the 26S proteasome in nuclear foci to facilitate quality control during replication [2]. Adeno-associated viruses (AAV) are helper-dependent parvoviruses that have evolved strategies to replicate efficiently by exploiting host cell co-infection by adenoviruses or herpesviruses [3]. The infectious pathway of wild type AAV and recombinant AAV vectors consists of multiple stages starting with cell surface receptor binding, followed by endocytosis, endosomal escape, nuclear import, second-strand synthesis, and subsequent expression of the vector-encoded transgene [4,5]. The post-entry steps leading to AAV transduction are particularly subject to restriction by cell intrinsic factors [6,7,8]. Studies have identified impaired AAV vector transduction due to inefficient nuclear import [6], uncoating of vector genomes [7], or second-strand synthesis [8,9]. Treatment with proteasome inhibitors has demonstrated improved transduction by AAV vectors [10,11], suggesting the involvement of proteasomal degradation pathways in restricting AAV transduction. Nevertheless, modest increases in accumulation of viral DNA following proteasomal inhibition cannot solely account for substantial increases in AAV transduction [12], and the underlying mechanism remains elusive. Other host factors such as the FKBP52 [13], Mre11/Rad50/Nbs1 complex [14,15], APOBEC3A [16] and more recently, TRIM19/promyelocytic leukemia protein (PML) [17] have been shown to inhibit AAV replication by blocking second-strand synthesis.

AAV has emerged as a promising vehicle to achieve long-term gene expression with low toxicity. Recombinant vectors based on naturally occurring AAV serotype capsids and libraries of engineered capsid mutants have demonstrated unique receptor usages and tissue tropisms, providing versatility for tissue-targeted gene expression [18,19,20,21,22]. For instance, AAV vectors with AAV serotype 9 (AAV9) capsid efficiently transduce cardiac tissues, while vectors with AAV2 capsid show efficient transduction of kidney cells [23,24,25]. Importantly, recent phase I and phase II clinical trials using AAV vectors have established their safety, in some cases, with notable clinical benefits [26,27,28,29,30], opening the door to AAV vector gene therapies for various human disease conditions. Currently, however, efficient gene transduction by AAV vectors typically requires high doses of vectors. This presents a major barrier for the widespread use of AAV vectors in the clinic, due to potential challenges in manufacturing clinical grade vectors for high dose studies as well as the increased risk of eliciting host immune responses or inducing insertional mutagenesis at high vector doses [31,32,33]. Improving AAV vector transduction efficiency would reduce vector doses required for efficient gene delivery, minimizing the risks associated with high dose AAV vectors. The current report is focused on the identification of novel host restriction factor(s) that limit expression from AAV vectors as well as proof-of-principle studies that would enable effective gene therapy with lower vector doses in clinical trials.

## Results

### Screening of the siRNA library for proteasomal pathway genes identifies PHF5A as a factor blocking AAV9 vector transduction

**Fig 1:**
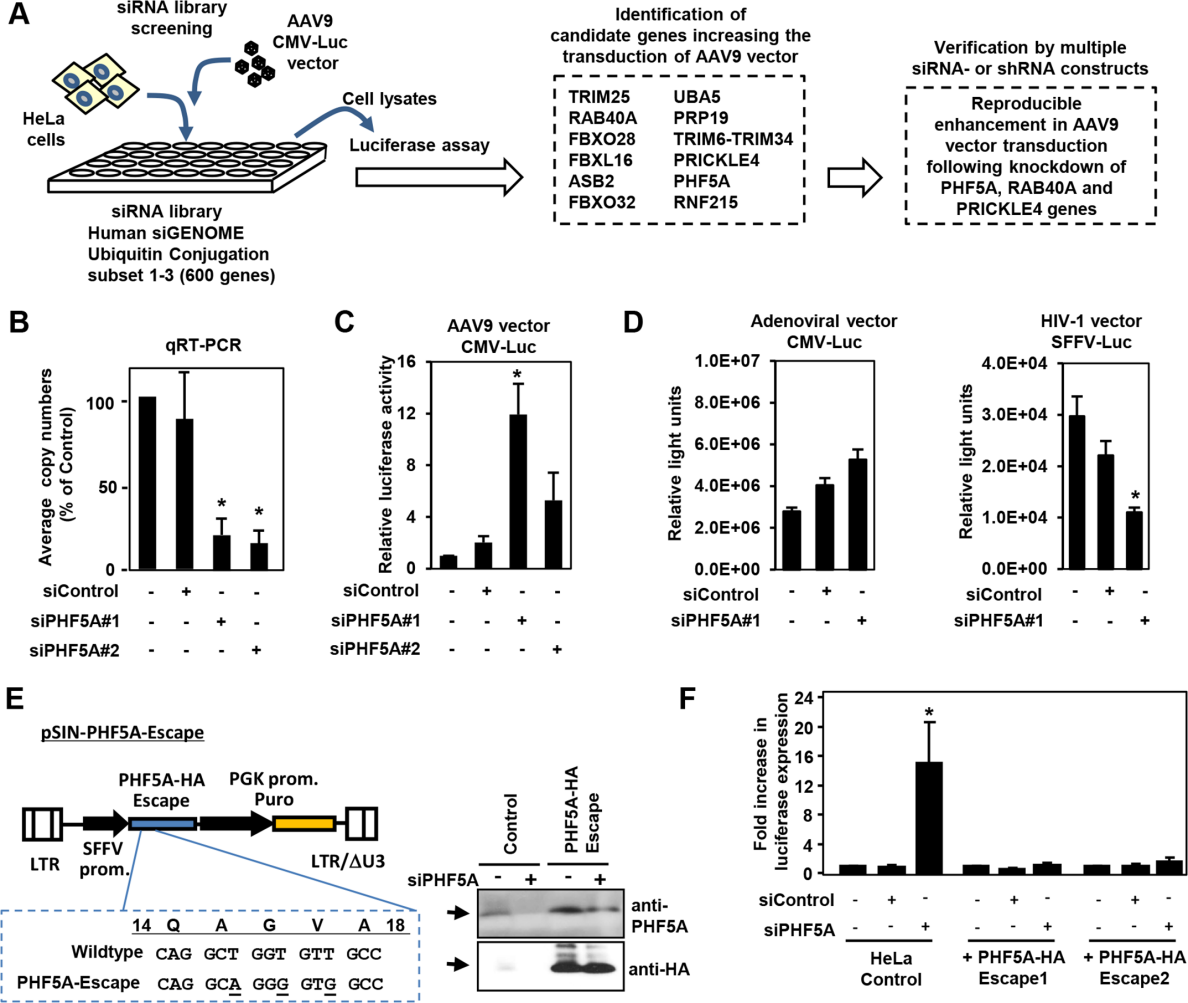
Screening of the siRNA library for proteasomal pathway genes identifies PHF5A as a factor blocking the transduction by AAV9 vector. **(A)** Screening of the siRNA library was carried out by reverse transfection of HeLa cells with siRNAs, followed by infection with luciferase-expressing AAV9 vectors (AAV9 CMV-Luc) at a multiplicity of infection (MOI) of 10^4^, and assessment of luciferase expression. Screening of the library identified 12 candidate genes that increased transduction by AAV9 vectors over 10-fold. Further studies were carried out in HeLa cells transfected/transduced with specific siRNAs or shRNA lentivectors for each of the 12 genes to verify the screening candidates. **(B)** Quantitative real-time RT-PCR was performed to determine the levels of PHF5A transcripts in cells treated with control or PHF5A siRNAs at 48 hours. **(C)** HeLa cells were transfected with control or PHF5A siRNAs for 24 hours, followed by infection with AAV9 CMV-Luc vectors (MOI 10^4^) for an additional 48 hours. The luciferase assay was performed in order to determine relative luciferase activities in treated cells. **(D)** Same as C, except that a luciferase-expressing adenoviral vector at an MOI of 3 x 10^2^ or an HIV-1-based lentiviral vector (MOI 0.3) were used to infect siRNA-treated HeLa cells. **(E)** Lentiviral vector pSIN-PHF5A-Escape with the PHF5A-HA Escape transgene was generated through introduction of three silent mutations in the PHF5A siRNA#1-targeted sequence. Western blotting was performed to verify the expression of the PHF5A-HA-Escape and its resistance to the PHF5A siRNA#1 treatment. Anti-PHF5A antibody was used to detect endogenous and over-expressed PHF5A-HA, while anti-HA antibody detected the HA-tagged PHF5A. **(F)** HeLa cell lines stably expressing the PHF5A-HA-Escape mutant were generated through lentiviral transduction of the escape mutant, followed by puromycin selection. Upon treatment with the PHF5A siRNA and AAV9 CMV-Luc vector (MOI 10^4^), luciferase expression was determined in control HeLa and PHF5A-HA-Escape-expressing HeLa cells. **(B-D, F)** Data are shown as averages of three independent experiments with error bars representing standard error of the mean. *p<0.05.

We screened an siRNA library, which covers 600 known and putative human genes in the ubiquitin and proteasome pathways, for AAV vector transduction. We identified 12 candidate genes (Fig 1A). Disruption of those genes in HeLa cells increased luciferase expression by an AAV9 vector, AAV9 CMV-Luc, over 10-fold (Fig 1A). Further verification with distinct siRNAs and lenti-shRNA vectors found disruption of PHF5A, RAB40A and PRICKLE4 reproducibly increased AAV9 transduction. Treatment of HeLa cells with two PHF5A siRNAs led to over 80% reduction in PHF5A transcripts (Fig 1B) and increased the transduction by AAV9 vectors up to 12-fold (Fig 1C). In contrast, disruption of PHF5A expression did not strongly enhance luciferase expression of adenoviral or HIV-based lentiviral vectors (Fig 1D). Similar results were observed upon disruption of RAB40A and PRICKLE4 (S1 Fig).

To rule out possible off-target effects of siRNA, we generated a lentiviral vector expressing an siRNA-resistant, HA-tagged PHF5A mutant, PHF5A-HA-Escape (Fig 1E). When endogenous PHF5A expression was disrupted by the PHF5A siRNA, two independent HeLa cell lines with stable PHF5A-HA-Escape expression (Fig 1E, right panel) did not show enhanced AAV9 vector transduction (Fig 1F). Thus, the increased expression from AAV9 vector by the PHF5A siRNA is PHF5A-specific, but not due to off-target effects.

### PHF5A blocks AAV vector transduction after second-strand synthesis

**Fig 2:**
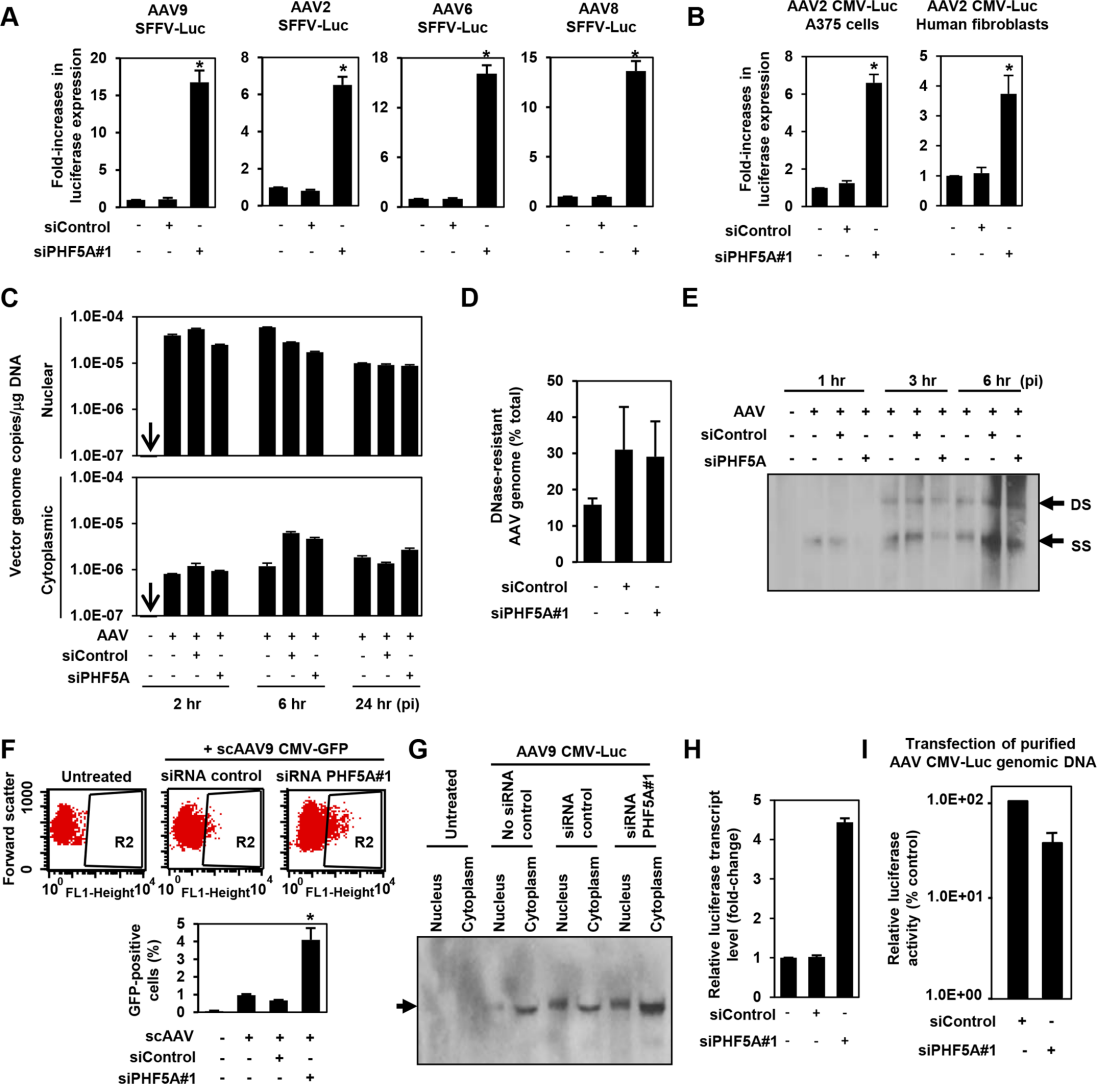
PHF5A blocks AAV vector transduction after second strand synthesis. **(A)** HeLa cells pre-treated with control or PHF5A siRNAs for 24hr were transduced with AAV9, 2, 6 or 8 vectors (MOI 10^4^) expressing luciferase under the control of the SFFV retroviral promoter with no splicing unit. Relative increase in luciferase expression was determined 48 hours p.i. Averages of three independent experiments were shown. Error bars represent standard error of the mean. **(B)** Melanoma A375 cells and primary human fibroblasts were pre-treated with siRNAs for 24 hours, followed by transduction with the AAV2 CMV-Luc vector (MOI 10^4^) for 48 hours. **(C)** HeLa cells were pre-treated with siRNAs for 24 hours and infected by the AAV9 CMV-Luc vector (MOI 8 x 10^4^). Total cytoplasmic and nuclear DNA were isolated and AAV luciferase vector genome copies were determined by quantitative real-time PCR at 2, 6 and 24 hours p.i. All samples were prepared in duplicate, and results represent the average of three separate experiments. **(D)** Same as C, but total DNA at 6 hours p.i. was used to determine total and DNase-resistant AAV genome copies and assess the percent DNase-resistant AAV genomes. Samples were in duplicate and results show the average of two independent experiments. **(E)** siRNA-treated HeLa cells were infected with AAV9 CMV-Luc vector (MOI 8 x 10^4^) for 1, 3 or 6 hours. Total nuclear DNA samples were used to detect the vector-derived single-stranded and double stranded monomers by Southern blotting. **(F)** HeLa cells were transfected with siRNAs for 24 hours, followed by infection with a GFP-expressing self-complementary (sc) AAV9 vector (MOI 2 x 10^4^) for 48 hours. Flow cytometry analysis was performed to quantify GFP-positive cell populations. The graph represents percentage of GFP-positive cells from the R2-gated population. **(G)** HeLa cells were pre-treated or untreated with siRNAs for 24 hours, followed by transduction with the AAV9 CMV-Luc vector (MOI 4 x 10^5^) for 36 hours. Nuclear and cytoplasmic RNA samples were subject to the Northern blotting analysis for detection of the luciferase transcripts. **(H)** HeLa cells were treated with no siRNA, control or PHF5A siRNAs for 24 hours and then transduced by AAV9 CMV-Luc (MOI 2 x 10^5^). Thirty-six hours p.i., cells were harvested and levels of luciferase transcripts were determined by RT-qPCR. **(I)** HeLa cells were pre-treated with control or PHF5A siRNAs for 24 hours, followed by transfection with purified AAV CMV-Luc genomic DNA (0.1 μg/well). Luciferase activities were determined at 48 hours p.i. *p<0.05.

The AAV CMV-Luc vector construct used in the library screening contained a human beta globin intron. To rule out the possibility of PHF5A modulating the CMV promoter activity or the intronic unit, we first replaced the CMV promoter and intron sequence in the AAV vector genome with an intron-less retroviral SFFV promoter. Disruption of PHF5A increased transduction by multiple AAV serotypes (Fig 2A), indicating that the PHF5A-mediated restriction was independent from internal promoters or receptors used by AAV vectors. Likewise, knocking down PHF5A was effective at increasing AAV vector transduction in other cell types, including A375 melanoma cells and primary cardiac fibroblasts (Fig 2B).

Next, we examined the influence of PHF5A ablation on multiple stages of AAV vector transduction. No notable effects were observed on AAV cellular or nuclear entry (Figs 2C and S2). Additionally, approximately 30% of total AAV DNA detected was DNase-resistant at 24 hours post infection (p.i.) (Fig 2D), indicating that PHF5A does not affect uncoating process of AAV vectors. Southern blot analysis demonstrated no notable increase in double-stranded-monomers in cells pretreated with the PHF5A siRNA (Fig 2E). Upon transduction with a GFP-expressing self-complementary AAV (scAAV) vector, which does not rely on second-strand synthesis for transgene expression, we found significant increases in GFP-expressing cell populations in HeLa cells treated with the PHF5A siRNA (Fig 2F). These results indicate that PHF5A blocks the process of AAV vector transduction after second-strand synthesis. We then explored the effects of PHF5A disruption on the transcription of AAV9 CMV-Luc vector. Northern blot analysis showed that pretreatment with the PHF5A siRNA increased the levels of luciferase-specific transcripts (Fig 2G and 2H), suggesting that PHF5A affects the step before translation. When HeLa cells were transfected with the AAV vector genome plasmid, pAAV CMV-Luc, or single-stranded AAV vector genomic DNA from purified AAV vector particles, PHF5A ablation caused no increase in luciferase expression of transfected viral genome (Fig 2I). Together, this suggests that PHF5A acts to restrict AAV vector transduction somewhere between AAV second-strand synthesis and the transcription of the AAV vector transgene. It also appears that PHF5A does not directly target AAV vector genome. Additionally, introduction of disruptive mutations in any of the three GATA-type zinc finger motifs in PHF5A led to the loss of anti-AAV activity (S3 Fig).

### The U2snRNP complex plays a key role in restricting AAV vector transduction

**Fig 3:**
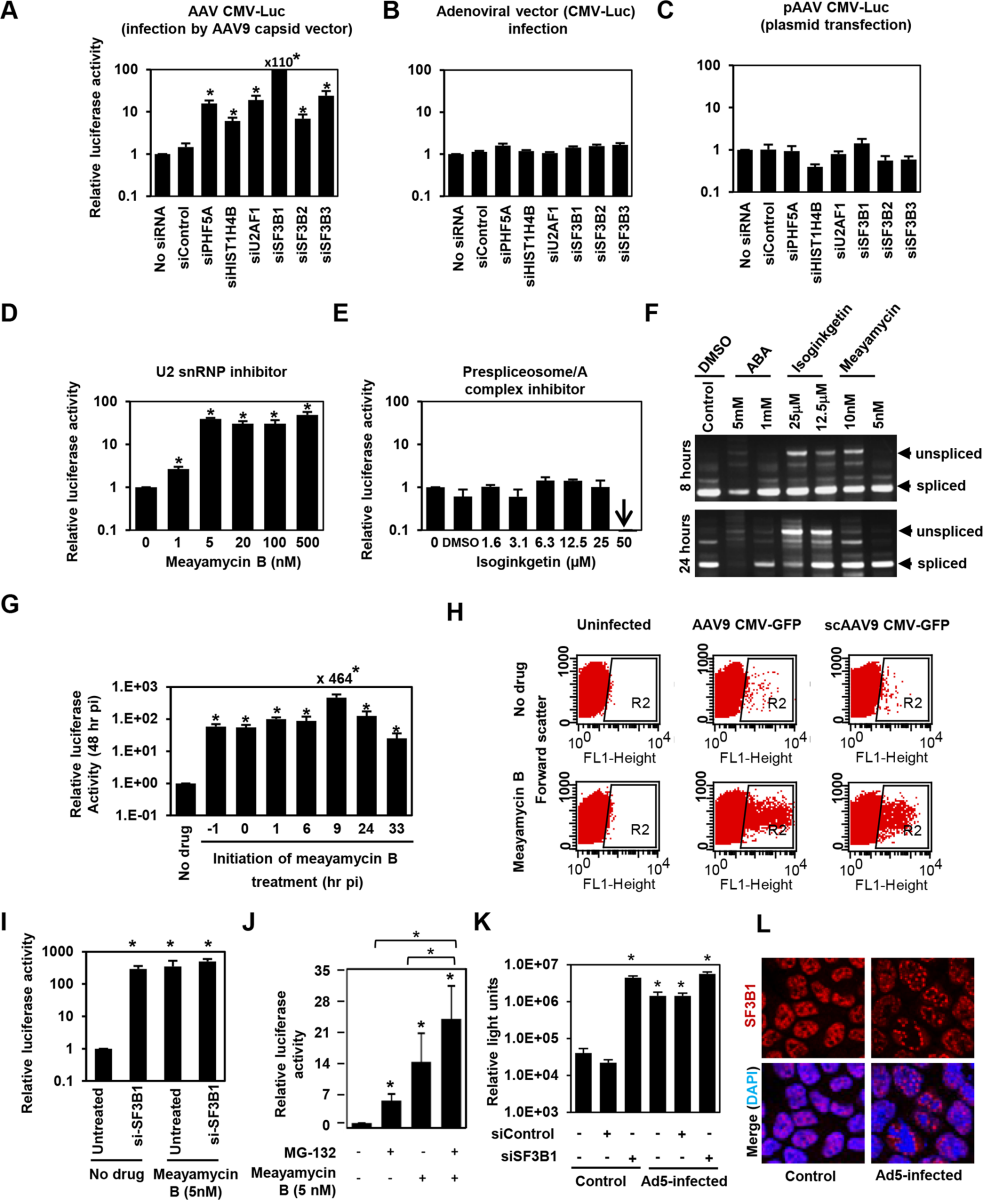
The U2snRNP complex plays the key role in restricting AAV vector transduction. **(A)** HeLa cells were transfected with control siRNA, or siRNAs targeting PHF5A, histone 4, U2AF1, SF3B1, SF3B2 and SF3B3 for 24 hours, followed by the AAV9 CMV-Luc vector transduction (MOI 10^4^). Relative luciferase expression was determined 48 hours p.i. **(B)** Same as A, but luciferase-expressing adenoviral vector was used to transduce siRNA-treated cells (MOI of 3 x 10^2^). **(C)** Same as A, except that the siRNA-treated cells were transfected with the vector genome plasmid, pAAV CMV-Luc (0.2 μg/well)for 48 hours. Note that this plasmid was used to generate the infectious AAV9 CMV-Luc vector used in (A). **(D)** HeLa cells were treated with increasing concentrations of U2 snRNP inhibitor, meayamycin B, followed by transduction with the AAV9 CMV-Luc vector (MOI 10^4^). Relative luciferase expression was determined 48 hours p.i. **(E)** Same as D, except that a prespliceosome/A complex inhibitor, Isoginkgetin, was used. **(F)** HeLa cells were treated with indicated spliceosome inhibitors for 8 and 24 hours and levels of unspliced and spliced cellular MAPT (microtubule associated protein tau) transcripts were determined by RT-PCR. **(G)** HeLa cells were treated with 20 nM meayamycin B at various time points before or after AAV9 CMV-Luc vector infection (MOI 10^4^). Relative luciferase expression was determined 48 hours p.i. **(H)** HeLa cells were infected by AAV9 CMV-GFP (MOI 10^3^) or scAAV9 CMV-GFP vectors (MOI 6 x 10^3^), followed by treatment with 20 nM meayamycin B at 8 hours p.i. Flow cytometry analysis was performed to see GFP-positive cell populations at 48 hours p.i. **(I)** Co-treatment of HeLa cells with SF3B1 siRNA and Meayamycin B. HeLa cells were treated with the siRNAs for 48 hr, followed by infection with AAV9 CMV-Luc (MOI 10^4^). At 9 hours p.i. Meayamycin B (5nM) was added, and cells were harvested for the luciferase assay 48 hours p.i. **(J)** Co-treatment of HeLa cells with MG-132 and Meayamycin B. 30 min prior to AAV infection cells were treated with MG-132. 9 hours after infection with AAV9 CMV-Luc (MOI 10^4^), cells were treated with meayamycin B, and harvested for luciferase assay 20 hours later. Due to notable toxicity of MG-132, we needed to harvest cells at this early time point. **(K)** Influence of dual treatment with human adenovirus 5 infection and SF3B1 disruption on AAV vector infection. HeLa cells were treated with control or SF3B1 siRNAs for 24 hours, followed by infection with AAV2 CMV-Luc (MOI 10^4^) or co-infection with AAV2 CMV-Luc and human adenovirus 5 (MOI 3 x 10^4^) for 48 hours. **(L)** Influence of adenovirus 5 infection on subcellular localization of SF3B1 in HeLa cells. HeLa cells were infected with human adenovirus 5 (MOI 10^4^) for 24 hours, and SF3B1 in control and infected HeLa cells was visualized by anti-SF3B1 antibody (red). Nuclei were counter-stained by DAPI (blue). (A-E, G, I, J and K) Samples were run in triplicate and results are the average of two independent experiments. *p<0.05.

PHF5A has been reported to interact with various proteins, including the U2 snRNP proteins, SF3B1, SF3B2, SF3B3 [34,35,36], U2AF1, ATP-dependent helicases EP400 and DDX1, and arginine-serine-rich domains of splicing factor SFRS5 [36]. Additionally, through co-immuno-precipitation of HA-tagged PHF5A, we identified potential PHF5A-interacting proteins, including FUS, EEF1, EEF2 and HIST1H4B. To further understand the underlying mechanism, we assessed the effects of disrupting those proteins on expression from AAV vectors. After verification of reduction in corresponding transcripts upon transfection of specific siRNAs (S4A Fig), siRNA-treated cells were infected with AAV9 CMV-Luc vectors at 24 hours post transfection, with luciferase activity assayed 48 hours p.i. Ablation of U2 snRNP components and U2 snRNP-associated factor (U2AF1) resulted in a substantial increase in luciferase activity relative to HeLa cells pre-treated with a control siRNA (Figs 3A and S4B). Disruption of HIST1H4B, one of histone H4 genes, also showed a modest increase, while ablation of other factors showed no notable effect. Of note, disruption of spliceosome proteins involved in other splicing steps, including SNRNP200 and PRPF31, essential factors for U4/U6-U5 formation and function, did not increase the AAV vector transduction (S4A and S4B Fig). These results suggest that PHF5A blocks AAV vector transduction through an interaction with U2 snRNP proteins and associated U2AF1, independently of cellular RNA spliceosome function. Similar to the effects of PHF5A knockdown, disruption of U2 snRNP components or U2AF1 did not enhance the luciferase expression from an adenoviral vector or a transfected AAV vector plasmid, pAAV CMV-Luc (Fig 3B and 3C). Taken together, we conclude that infectious AAV particles and all steps in intracellular trafficking pathway are essential for the restriction of transduction by U2 snRNP and associated proteins.

To further confirm the role of U2 snRNP proteins in the restriction of AAV vectors, we assessed the influence of pharmacological inhibition of U2 snRNP on expression from AAV vectors. One drug we employed was meayamycin B, a potent SF3B1 inhibitor, synthesized according to the literature [37]. When HeLa cells were pre-treated with this drug at an increasing dose 3 hours before AAV9 vector infection, dose-dependent increases (up to 49-fold) in relative luciferase activity were seen (Fig 3D). As we reported previously, treatment of HeLa cells with over 20 nM of meayamycin B for two days showed cytostatic effects [37]. A related SF3B1 inhibitor, meayamycin, also demonstrated a substantial increase in AAV transduction (S4C Fig). In contrast, other drugs reported to block other splicing steps, including isoginkgetin at the prespliceosome/A complex stage [38] and 3-Aminophenylboronic acid (ABA) at the second stage (excision of the lariat intron), did not show notable increases in AAV vector transduction (Figs 3E and S4D) although isoginkgetin blocked cellular mRNA processing (Fig 3F). Of note, a low dose (5 nM) meayamycin B treatment substantially enhanced AAV vector transduction without strongly affecting mRNA splicing (Fig 3D and 3F), indicating that inhibition of the general splicing process is not necessary to enhance AAV vector transduction. Additionally, we found that pretreatment with the drug is not needed in order for it to enhance AAV vector infection (Fig 3G). The largest increase in luciferase activity (464-fold) was observed when cells were treated by meayamycin B 9 hours p.i. In contrast, treatment at 33 hours post p.i. showed relatively weak effects. To further map the optimal timing of U2 snRNP inhibition for AAV vector transduction, we treated AAV2 and AAV9 vector-infected HeLa cells with 5 nM meayamycin B at various time points and duration, and assessed luciferase activity at 3 days p.i. Treating with meayamycin B 3 hours p.i. and washing cells 1, 2 or 3 days after receiving the drug resulted in similarly high levels of enhanced luciferase expression (S4E Fig). Washout of meayamycin B for 48 hours after 3–24 hours of treatment did not strongly compromise its effects on AAV vector transduction. In contrast, the effects of meayamycin B on AAV vector transduction were impaired when drug was added either 24 or 48 hours p.i. (S4E Fig). Thus, optimal enhancement of AAV vector transduction requires initiation of U2 snRNP inhibition prior to 24 hours post AAV vector infection. This indicates that U2 snRNP blocks AAV vectors at a particular post-entry step of viral infection, likely occurring before 24 hours p.i. Similar to PHF5A disruption, meayamycin B also enhanced the transduction by both single-stranded AAV and scAAV vectors through increasing the number as well as the fluorescent intensity of GFP-positive cells (Fig 3H). In addition, dual treatments with meayamycin B and PHF5A or SF3B1 siRNAs showed no additional impact on the AAV vector transduction (Figs 3I and S4F), verifying that meayamycin B and PHF5A/SF3B1 target a common pathway. On the other hand, dual treatment with a proteasomal inhibitor MG132 and meayamycin B showed a synergistic effect (Fig 3J), indicating that the U2 snRNP proteins block AAV restriction independently from the proteasomal pathway. We also tested the influence of adenovirus co-infection on the U2 snRNP-mediated restriction of AAV vectors. Although human adenovirus 5 (Ad5) co-infection alone enhanced AAV2 transduction 30-fold (Fig 3J), dual treatments of Ad5 co-infection and SF3B1 knockdown showed minimal additive effects on AAV transduction (Fig 3K). Similar results were observed with meayamycin and Ad5 dual treatments (S4G Fig). These results strongly suggest that Ad5-mediated activation of AAV vector transduction is through U2 snRNP inhibition. Indeed, when the influence of Ad5 infection on subcellular localization of U2 snRNP was determined, Ad5 infection showed notable PHF5A and SF3B1 displacement in HeLa cells (Figs 3L and S4H). On the other hand, meayamycin B treatment failed to increase AAV vector production, or rescue AAV vector production in the absence of the adenovirus helper plasmid (S4I Fig), suggesting that U2 snRNP inhibition is not sufficient to provide adenoviral helper function during AAV production.

### PHF5A and U2 snRNP component SF3B1 interact with AAV capsid

**Fig 4:**
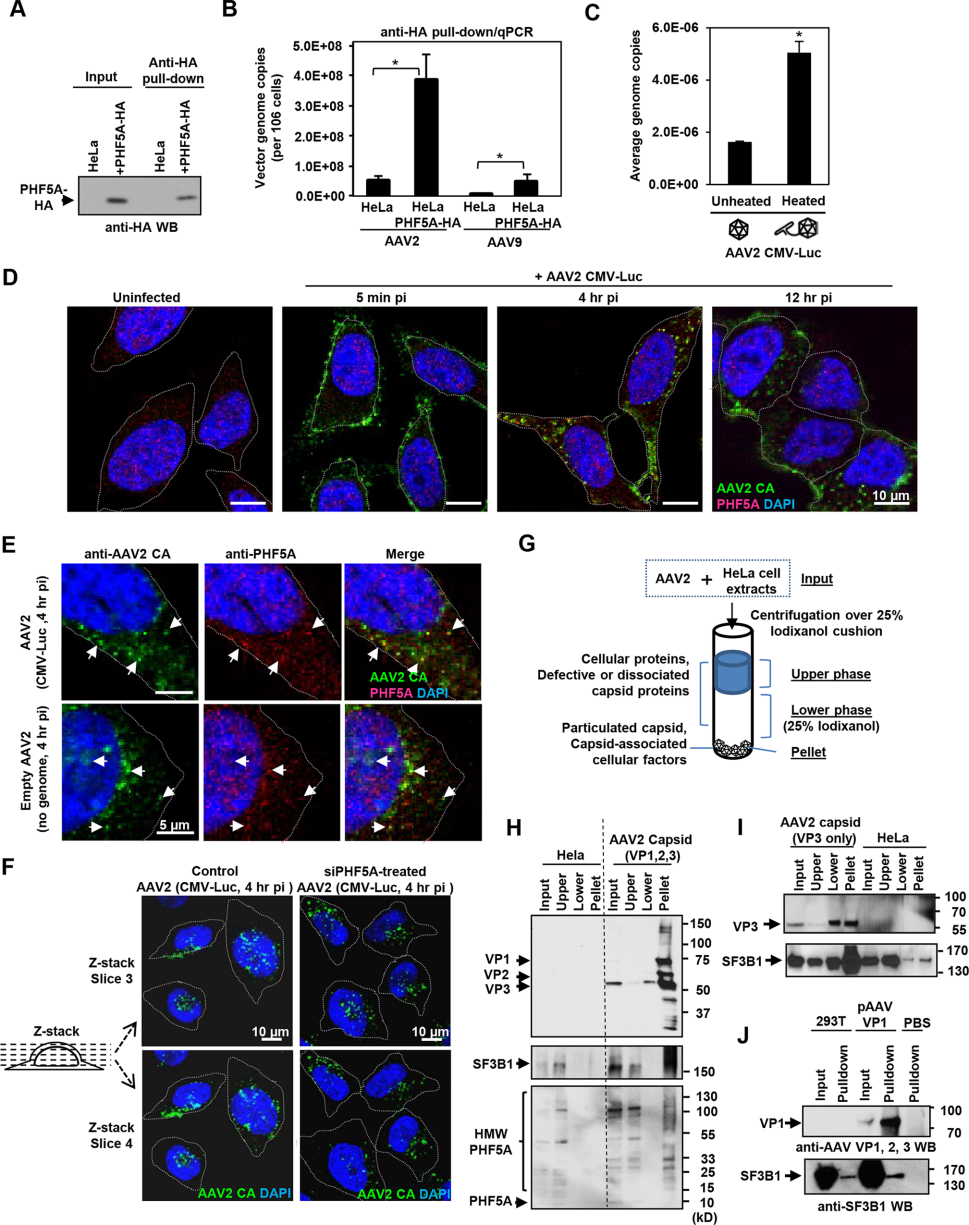
PHF5A and U2 snRNP component SF3B1 interact with AAV capsid. **(A)** HeLa or PHF5A-HA-expressing cell lysates were used to pull-down the HA-tagged PHF5A by anti-HA agarose beads. After 15 washes, the HA-tagged PHF5A was detected by anti-HA antibody. **(B)** Control or PHF5A-HA-expressing HeLa cells were transduced by AAV2 and AAV9 CMV-Luc vectors (MOI 4 x 10^5^) and total AAV genome copies in the HA pulldown were determined by quantitative real-time PCR. **(C)** AAV2 CMV-Luc vector (3 x 10^10^ genome copies) was unheated or preheated for 30 min at 65°C. PHF5A-HA-over-expressing HeLa cell lysates were then incubated with vectors for 1 hour at 4°C, followed by pulldown of PHF5A-HA. AAV vector genome copies in the precipitates were determined by quantitative real-time PCR. **(D)** HeLa cells were infected with the AAV2 CMV-Luc vector (4 x 10^10^ genome copies/well) for 5 min, 4 or 12 hours. Confocal microscopy analysis was performed to detect the subcellular localizations of AAV vector particles (green) and PHF5A (red). Nuclei were counterstained by DAPI (blue). **(E)** HeLa cells were infected with AAV2 CMV-Luc vectors (4 x 10^10^ genome copies/well) or equivalent amounts of empty AAV2 vectors for 4 hours, and cells were analyzed for co-localization of AAV2 capsid and endogenous PHF5A signals. Prominent co-localized signals were indicated by white arrows. **(F)** HeLa cells were treated with the PHF5A siRNA for 24 hours, followed by transduction with the AAV2 vector as in E for 4 hours. AAV2 vector particles were detected by anti-AAV2 capsid A20 antibody, and the patterns of cytoplasmic and nuclear accumulations of AAV2 vector particles were compared between control and PHF5A-ablated cells. Representative Z-stack images of the middle sections (slices 3 and 4) from control and PHF5A knockdown cells are shown. **(G)** Schematic representation for the iodixanol cushion method to enrich cellular factors interacting with particulated AAV capsids. **(H)** HeLa cell lysates were incubated with AAV2 CMV-Luc vectors (5 x 10^10^ genome copies) for 1 hour at 4°C. After centrifugation over 25% iodixanol, three layers (the upper phase, lower phase, and pellet) were separately harvested for Western blotting. AAV capsid proteins VP1, 2 and 3, phospho-SF3B1, and endogenous PHF5A were detected by A20, anti-SF3B1, and anti-PHF5A antibodies, respectively. **(I)** Same as H for AAV capsid proteins, except that empty AAV2 VP3 only capsids were used for SF3B1 co-precipitation. **(J)** Control or AAV VP1-over-expressing 293T cell lysates were used to pull-down the AAV VP1 protein by A20 antibody. After 15 washes, the pellets were probed for SF3B1 enrichment by anti-SF3B1 antibody.

Next, we assessed the interaction between PHF5A and AAV vector components. Upon pull-down of HA-tagged PHF5A from AAV vector-infected cells (Figs 4A and S5), total DNA in the pellets was assessed for AAV vector genomes. Quantitative real-time PCR detected significantly more AAV vector genome DNA in the HA pulldown from PHF5A-HA-over-expressing cells as opposed to control cells (Fig 4B). We also tested the influence of heat-mediated conformational changes of viral capsids, which lead to the exposure of the hidden VP1 N-terminal and viral genomic DNA [39,40], on the interaction with PHF5A. A three-fold increase in AAV genome copies was detected in the HeLa-PHF5A-HA pulldown when the cell lysates were incubated with pre-heated AAV2 CMV-Luc vector than non-heated vector (Fig 4C). Those data indicate interaction of PHF5A with AAV vector genome, directly or indirectly.

Next, immunohistochemistry was performed in order to identify the subcellular localization of PHF5A and AAV capsid. The A20 anti-AAV2 capsid antibody was used to detect AAV capsids (Fig 4D). The majority of AAV capsid signals were found in the cytoplasm at 4 and 12 hours p.i. (Fig 4D). Although endogenous PHF5A was predominantly found in the nucleus, especially in the nucleoli, of uninfected cells (Fig 4D), a notable increase in cytoplasmic PHF5A signals was found in AAV2 vector-infected cells at 1 and 4 hours p.i. (S6A Fig). Of note, AAV2 capsid signals frequently co-localized with the cytoplasmic PHF5A body signals (Fig 4E). When HeLa cells were exposed to an empty AAV2 vector, similar cytoplasmic recruitment of PHF5A to AAV2 capsids was also evident (Fig 4E, lower panels), suggesting that the PHF5A translocation (or de novo recruitment in the endosome) to AAV2 capsids is independent of AAV vector genome. Analysis of Z-stack images of AAV-infected cells at 4 hours p.i. showed comparable perinuclear and nuclear accumulation of AAV capsids between control and the PHF5A-siRNA treatment, further supporting that PHF5A does not affect AAV vector trafficking and nuclear import (Figs 4F and S7).

When the subcellular localization of the major U2 snRNP component, SF3B1, was assessed, nuclear speckles in the nucleoli and a diffuse cytoplasmic signal were observed (S8A Fig). Although the widespread SF3B1 signals often overlapped with the AAV2 capsid signals, the diffuse cytoplasmic signal made it difficult to verify co-localization with AAV2 vector particles (S8B Fig). To validate the AAV2 capsid and SF3B1 interaction, we employed the gradient technique to separate free-SF3B1 forms from particulated AAV capsid-associated SF3B1 (Fig 4G), which was used to determine the interaction between retroviral capsids and a cytoplasmic retroviral restriction factor TRIM5alpha [41]. Upon ultracentrifugation through the 25% iodixanol layer, AAV2 capsid proteins VP1, VP2 and VP3 (87, 72, 62 kDa) were detected in the pellets (Fig 4H). When the same samples were probed for SF3B1 (175 kDa), endogenous SF3B1 was seen in the input and upper layer samples of untreated HeLa cells (Fig 4H). In AAV2-treated lysates, additional SF3B1 bands were seen in the pellet (Fig 4H, S8C and S8D Fig). When the co-precipitation of PHF5A with AAV2 capsid was assessed, no intact PHF5A protein bands (15 kDa) were detected after incubation at 4°C for 1 hour, likely due to its instability (Fig 4H). Instead, multiple high molecular weight signals were detected in the input and top layer samples by anti-PHF5A and anti-HA antibodies (Figs 4H and S8D). Notably, high molecular weight PHF5A signals were detected in the pellet of AAV2-treated samples but not in the pellet of untreated samples. It is possible that PHF5A is modified (or modifies other proteins) upon interaction with AAV components. To further map the responsible region for the interaction between SF3B1 and AAV2 capsid, we performed the same experiments using empty AAV capsid, made with the VP3 protein alone. The VP3-only capsid was able to enrich SF3B1 in the pellets (Fig 4I). In contrast, multiple attempts to enrich SF3B1 or PHF5A through pulling down non-assembled VP1 proteins were unsuccessful (Fig 4J). These results suggest that U2 snRNP proteins interact with the AAV2 capsid structure within the VP3 region, but not AAV2 vector genomic DNA or non-assembled AAV2 VP1 protein.

### Meayamycin B increases AAV vector transduction of clinically relevant cell types

**Fig 5:**
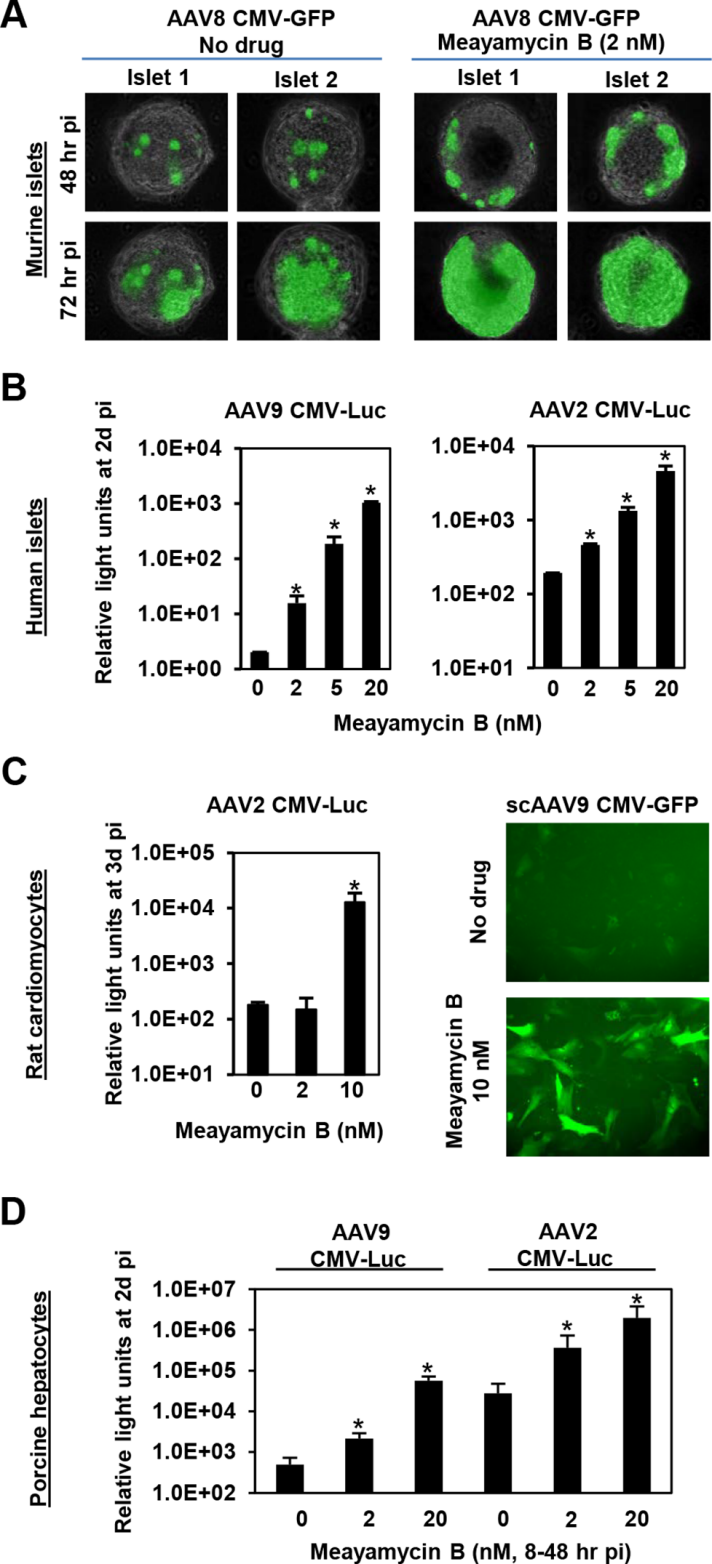
Meayamycin B increases AAV vector transduction of clinically relevant cell types. **(A)** Primary mouse islets were infected with AAV8 CMV-GFP in the presence or absence of 2 nM meayamycin B, and GFP expression was monitored for three days. **(B)** Primary human islets were treated with AAV2 or AAV9 CMV-Luc vectors for 7 hours and then treated with 0, 2, 5 or 10 nM meayamycin B. Luciferase expression was analyzed 48 hours p.i. **(C)** Neonatal rat cardiomyocytes were infected with AAV2 CMV-Luc or scAAV9 CMV-GFP vectors and treated with meayamycin B, 3 hours p.i. Luciferase activity was measured 3 days p.i., while GFP expression was monitored at 5 days p.i. **(D)** Porcine hepatocytes were infected with AAV2 or AAV9 CMV-Luc vectors for 8 hours, virus was then removed and cells were treated with 0, 2, or 20 nM meayamycin B. Cells were harvested 48 hours p.i. for the luciferase assay. In A-D, an MOI of 10^4^ was used.

Finally, we tested the ability of meayamycin B to boost AAV transduction in various cell types, relevant to gene therapy applications. When primary pancreatic islets were transduced with AAV8 CMV-GFP and treated with 2 nM meayamycin B 3 hours p.i., there were increased numbers of GFP expressing cells in drug treated mouse islets (Fig 5A). When primary human pancreatic islets were infected with AAV2 or AAV9 CMV-Luc vectors and treated with 0, 2, 5, or 20 nM meayamycin B at 7 hours p.i., we found dose-dependent increases in luciferase expression in AAV2 and AAV9 infected cells (Fig 5B). Likewise, meayamycin B treatment increased AAV2 and AAV9 transduction of primary neonatal rat cardiomyocytes as well as porcine hepatocytes (Fig 5C and 5D). These results demonstrate that meayamycin B enhances AAV vector transduction of a variety of cell types from different host species. Although we typically observed no notable toxicity in primary cells treated with 5 nM meayamycin B, prolonged treatment with over 10 nM meayamycin B showed anti-proliferative effects as we reported previously [42]. Since meayamycin B is rapidly cleared from circulation by unknown mechanism(s) [42], we were unable to evaluate drug doses high enough to test the impact on AAV vector transduction *in vivo*.

## Discussion

Here, we have demonstrated that PHF5A and U2 snRNP proteins, such as SF3B1, restrict AAV vector transduction through recognition of incoming AAV capsids. Of particular relevance to gene therapy applications, genetic and pharmacological inhibition of PHF5A or U2 snRNP-associated proteins strongly increased the transduction efficiency of AAV vectors. Thus, transient suppression of U2 snRNP or designing AAV vectors to avoid this restriction can provide a novel strategy to achieve efficient AAV vector transduction with reduced vector doses, which in turn could lead to improved safety profiles for AAV-mediated gene therapy applications.

Several strategies have demonstrated the potential to enhance AAV vector transduction efficiency, including treatments with genotoxic agents [43,44,45], adenoviral E1b55k/E4orf6 proteins [14], a specific EGFR protein tyrosine kinase inhibitor (Tyrphostin-23) [46], and proteasome inhibitors [10,11]. A major effect of the genotoxic treatments, such as hydroxyurea and topoisomerase inhibitors, is improved double-strand synthesis of the input vector genome [8,9,47]. In contrast, the adenoviral proteins degrade the cellular Mre11 repair complex (MRN) to promote AAV vector transduction as well as provide crucial helper functions for wild-type AAV replication [14], although a recent study suggests a role for MRN in gene expression [15]. Tryphostin-23 dephosphorylates FKBP52, a protein binding to the viral single-strand DNA, and improves viral second-strand DNA synthesis [46] and intracellular trafficking of AAV vectors [48]. Importantly, Tryphostin-23 does not show a synergistic effect with the proteasome inhibitor MG132, suggesting that both drugs target a common step of AAV vector transduction [12,48]. We found U2 snRNP inhibition had no notable effect on AAV vector second strand synthesis. Although we initiated the screening of the study using a commercially available siRNA library targeting known and putative proteasomal pathway proteins, MG132 treatment showed an additive effect with SF3B1 inhibition, suggesting U2 snRNP inhibition and MG132 work on distinct pathways. In contrast, dual treatments with Ad5 co-infection and SF3B1 inhibition showed no additive effect, suggesting a common target shared by Ad5 and SF3B1 inhibition. Since Ad5 infection induced PHF5A and SF3B1 displacement, it is plausible that Ad5 co-infection increases expression from AAV vectors, at least in part, through U2 snRNP inhibition.

Dissection of the mechanism by which PHF5A and U2 snRNP components block AAV vector transduction might allow rational design of next generation AAV vectors that can potentially circumvent this host restriction machinery. Based on the following observations, we conclude that U2 snRNP restricts AAVs at an early stage of infection. First, optimal enhancement of AAV vector transduction required U2 snRNP inhibition at an early time point post AAV vector infection (3–24 hours p.i.), while washout of a U2 snRNP inhibitor for the following 2 days did not impair the effects. Thus, short-term U2 snRNP suppression appears to change the fate of AAV vectors up to 2 days post-incubation. Secondly, U2 snRNP inhibition showed no enhancing effect when purified AAV vector genomic DNA or AAV vector genome plasmids were introduced by transfection. Third, although PHF5A was predominantly found in the nuclei in uninfected HeLa cells, we found frequent co-localization of AAV vector particles and PHF5A signals, both in the nucleus and the cytoplasm of cells exposed to AAV vectors. We speculate that newly synthesized PHF5A likely interacts with incoming AAV capsids in the cytoplasm. Finally, pull-down and capsid co-precipitation assays using AAV vectors and empty AAV particles indicate that PHF5A and SF3B1 interact with the capsid, likely mediated by domains within the VP3 region. Of note, the use of heated AAV particles, which leads to exposure of the hidden VP1 N-terminal and viral genome release [39,40,49], increased co-precipitation of capsid-associated AAV genome by PHF5A pull-down, or SF3B1 co-precipitation by the capsid. Consistent with these observations, previous studies have demonstrated the majority of viral DNA can remain associated with the capsid upon thermally induced DNA release [40]. A recent study has also implicated AAV capsid proteins in playing a role in second strand synthesis as well as the transcription of vector genomes [50], supporting prolonged association of AAV capsid proteins with vector genomes at the time of transcription in the nucleus. Taken together, our results strongly support the notion that direct interaction of PHF5A and U2 snRNP components in a cooperative fashion with conformationally altered AAV capsids and the exposed vector genome blocks subsequent transcription.

Although the exact mechanism is currently under investigation, one potential path being explored hinges on the involvement of U2 snRNP proteins in chromatin regulation. For instance, Isono et al. [51] have reported the essential role of SF3B1 (and likely other U2 snRNP proteins) in mammalian polycomb-mediated epigenetic silencing of homeotic genes. Sudemycin E, a U2 snRNP inhibitor, has also been shown to cause changes in histone modifications [52]. SF3B1 and SF3B2 are also found to associate with the histone H3 tails [53]. The ability of PHF5A and SF3B proteins to recruit additional factors to the AAV capsid and its associated vector genome is currently unknown, but if true these findings would provide further insight into the mechanism of host restriction. Further insight into the latter mechanism can potentially be derived from earlier reports that suggest that the splicing machinery is significantly remodeled during host cell infection by helper viruses such as adenoviruses [54]. As outlined earlier, the spatial organization of host splicing factors into distinct clusters within the nucleus appears to be regulated during adenoviral infection [1,55]. Similarly, herpes simplex virus infection induces snRNP-containing bodies [56] through interaction between IE63 protein (ICP27) with SF3B2 (SAP145) [57]. Thus, it is tempting to speculate that wild type AAV might have evolved to exploit the mislocalization/sequestration of splicing factors during helper virus co-infection, while recombinant AAV are unable to evade such host restriction factors in the absence of helper viruses. It is also possible that U2 snRNP plays a role as a broad spectrum antiviral factor, while helper viruses have evolved to counteract this restriction through sequestration of snRNP proteins.

Based on the aforementioned reasons, our current working model is the U2 snRNP recognition of incoming AAV capsid, leading to subsequent block of AAV transcription. However, some observations suggest potential U2 snRNP-mediated AAV restriction at a late stage of transduction. For instance, at very late time points in infection in cell cultures, there was still a substantial enhancement in AAV vector (100-fold at 24 hr and 20-fold at 33 hr p.i.). At this late time point, most of the genomes are considered to be in the nucleus, and it is less likely that U2 snRNP can target incoming AAV capsid. One plausible explanation is that U2 snRNP can also target AAV genome-associated capsid in the nucleus for blocking AAV vector expression. Another point is on our Northern blot analysis of vector transcripts upon PHF5A knockdown. We found a notable increase in cytoplasmic AAV transcripts, but lesser degree in nuclear transcripts. Thus it remains possible that U2 snRNP can also target the nuclear export/cytoplasmic accumulation of AAV transcripts. Another caveat of our experimental system was the use of rapidly dividing cells, where some, or even the majority, of vector genomes can be lost at later time points.

In addition to mechanistic analysis, we have compared FR901464 analogs and herboxidiene, and have identified that meayamycin B is the most potent SF3B1 inhibitor [58]. Importantly, treatment with meayamycin B substantially enhanced AAV vector transduction in various clinically relevant cell types, including primary cardiomyocytes, pancreatic islets and hepatocytes. Thus, pharmacological inhibition of U2 snRNP components may provide a novel strategy to improve AAV vector transduction. Unfortunately, however, intravenous administration of meayamycin B leads to rapid clearance, likely due to absorption, distribution, metabolism and/or excretion [42]. Additionally Meayamycin B also has a potent anti-proliferative effect at higher doses [37,58]. These features present a barrier to immediate *in vivo* applications of meayamycin B for improved AAV gene delivery. Nevertheless, since a low dose meayamycin B substantially increased AAV vector transduction without strongly affecting host RNA splicing *in vitro*, designing a novel U2 snRNP inhibitor with reduced cytostatic effects and *in vivo* stability may allow co-administration of the inhibitor with AAV vectors for improved AAV gene therapy with reduced vector doses.

In conclusion, we demonstrate that the U2 snRNP spliceosome inhibits AAV vector transduction and genetic/chemical modulation of this machinery improves transduction efficiency. This finding may lead to approaches that might help reduce AAV vector doses in clinical applications. Further understanding the underlying mechanism would provide novel insights into host-virus interactions and could inform the rational or combinatorial design of next generation AAV vectors with improved transduction efficiency and safety profile.

## Materials and Methods

### Ethics statement

Primary human islets were obtained through the Integrated Islet Distribution Program (IIDP) and the use of the cells was approved by the Mayo Institutional Review Board (IRB10429). All animal experiments were conducted according to the National Institute of Health guidelines and approved by the Institutional Animal Care and Use Committee (IACUC A33214 and IACUC A9014).

### Plasmids

pAAV-CMV-Luc vector genome construct, which drives firefly luciferase expression by a CMV internal promoter followed by the human beta globin intron, was described previously [24]. pAAV-SFFV-Luc was generated by replacing the CMV promoter and the beta globin intron region by Mlu1-BamHI with the intron-less SFFV retroviral promoter from a lentiviral vector plasmid, pSIN-Luc. The HA-tagged wildtype PHF5A-expressing lentiviral vector, pSIN-PHF5A-HA, was constructed by amplifying the human PHF5A ORF (GenBank accession number BC075808) in pCMV-SPORT6 (OpenBiosystems, MHS1010-97228317) by primers with a 3’ hemagglutinin (HA) tag, followed by cloning into the BamHI and NotI sites of a pHR-SIN CSGW PGK Puro (gift from Prof. Paul J. Lehner). Site-directed mutagenesis was performed to generate pSIN-PHF5A-Escape, with three point mutations in the PHF5A siRNA #1 targeting site. Further site-directed mutagenesis was performed to generate zinc finger mutant vectors, pSIN-PHF5A-HA-Esc-C46A/C49A, C58A/C61A and C72A/C75A (zinc fingers 3, 1 and 2 mutants, respectively). Primers used in the cloning are 5’-BamHI-F, 5'-GTCGGATCCGCCACCATGGCTAAACATCATCCTGA; 3’-NotI-HA-R, 5'-GGAGCGGCCGCTCAGGCGTAGTCAGGCACGTCGTAAGGATACCTCTTCTTGAAGCCGTATT; PHF5A-Escape-F, 5'-GCCGCAAGCAGGC**A**GG**G**GT**G**GCCATCGGAAG; PHF5A-Escape-R, 5'-CTTCCGATGGCCACCCCTGCCTGCTTGCGGC; PHF5A-ZF1m-F, 5'-ACCAGGGGCGCGCT**GT**GATC**GC**TGGAGGACCTGGGG; PHF5A-ZF1m-R, 5'-CCCCAGGTCCTCCAGCGATCACAGCGCGCCCCTGGT; PHF5A-ZF2m-F, 5'-GGTCTCTGATGCCTATTAT**GC**TAAGGAG**GC**CACCATCCAGG; PHF5A-ZF2m-R, 5'-CCTGGATGGTGGCCTCCTTAGCATAATAGGCATCAGAGACC; PHF5A-ZF3m-F, 5'-GGTGCGCATA**GC**TGATGAG**GC**TAACTATGGATCTTACCAG; PHF5A-ZF3m-R, 5'-CTGGTAAGATCCATAGTTAGCCTCATCAGCTATGCGCACC.

### Cells

HeLa (ATCC), 293T (ATCC), A375 (ATCC) and primary human cardiac fibroblast cells (ScienCell Research Laboratories) were cultured in Dulbecco’s modified Eagle’s medium containing 10% fetal bovine serum (FBS) (GIBCO) and antibiotics (penicillin 100 U/mL and streptomycin 100 μg/mL) (Corning Cellgro). HeLa cells stably expressing a series of PHF5A mutants were generated by transduction of corresponding lentiviral vector, followed by puromycin selection. Human islets were obtained through Integrated Islet Distribution Program and cultured in RPMI1640 medium supplemented with 10% FBS and antibiotics. Murine islets were harvested through intraductal collagenase perfusion and enzymatic digestion of the pancreas as previously described [59], and maintained in RPMI1640 medium supplemented with 10% FBS and antibiotics. Porcine hepatocytes were isolated from 15–20 kg pigs by a 2-step collagenase perfusion technique as previously described [60], and cultured in DMEM medium supplemented with 10% FBS, 10mM HEPES and antibiotics. Primary cardiomyocytes were isolated from newborn Dahl salt-sensitive rats using the Neonatal Cardiomyocytes Isolation System (Worthington, Lakewood, NJ) according to the manufacturer’s instruction. Beating cardiomyocytes were plated in gelatin/fibronectin-coated plates in DMEM medium supplemented with 10% FBS.

### Viral vectors

Helper-free AAV vectors were produced by transfection of three plasmids as described previously [61]. Briefly, 293T cells were transfected with three plasmids, including pHelper (Stratagene), one of the RepCap-expression plasmids (pRep2Cap2, pRep2Cap6, pRep2Cap9, or pRep2Cap8, kindly provided by Dr. James Wilson) and a transfer vector plasmid (pAAV-CMV-Luc, pAAV-SFFV-Luc, pAAV-CMV-Emerald GFP, or pScAAV-CMV-GFP [62]. pScAAV-CMV-GFP plasmid was kindly provided by Dr. R Jude Samulski through the National Gene Vector Biorepository. The resulting vectors were gradient purified using iodixanol (Optiprep Density Gradient Medium, SigmaAldrich), desalted and concentrated using Amicon Ultra-15 100k filtration (Amicon, Billerica, MA, USA) and resuspended in PBS. The genome copies (gc) of concentrated AAV vector stocks were determined by quantitative PCR as described previously [24]. Luciferase- or shRNA-carrying lentiviral vectors were produced as described previously [63]. Human adenovirus 5 (ATCC VR1516) was purchased from ATCC. Unless otherwise stated, no helper virus co-infection was used during AAV vector transduction.

### siRNA library

Human siGENOME Ubiquitin Conjugation Subsets #1 (89 genes), #2 (115 genes) and #3 (396 genes), a SMARTpool siRNA Library in Reverse Transfection Format (RTF) covering 600 gene targets, were purchased from Thermo Fisher Scientific. According to the provided RTF protocol, 5,000 cells/well HeLa cells were seeded, followed by AAV9 CMV-Luc infection at a multiplicity of infection of 100 (gc/cell). 48 hours after infection, luciferase assay was performed using the ONE-Glo Luciferase Assay System (Promega).

### siRNA and shRNA treatment and luciferase assay

HeLa cells were seeded in a 96-well plate at 5,000 cells/well for one day. Cells were then transfected with 0.5 μL of 10 μM siRNA using DharmaFECT Transfection Reagents (Thermo Fisher Scientific) according to the manufacturer’s instruction. Following siRNAs were used; control siRNA (siKrt1 5 SI02636732 from Qiagen), siPHF5A#1 and #2 (PHF5A 6 SI04210892 and 7 SI04310621) from Qiagen, siGenome Smart Pool siRNAs for PHF5A-interacting proteins;—siHIST1H4B (NM_003544, cat# M-011463-00), siU2AF1 (NM_001025203, cat# M-012325-01), siSF3B1 (NM_001005526, cat# M-020061-02), siSF3B2 (NM_006842, cat# M-026599-03), siSF3B3 (NM_012426, cat# M-020085-01). Twenty four hours post transfection, cells were infected with luciferase- or GFP-expressing vectors for 2 days.

### Splicing inhibitors

Meayamycin B was described previously [58]. Isoginkgetin was purchased from Millipore and resuspended in DMSO. 3-Aminophenylboronic acid was purchased from Sigma and resuspended in DMSO.

### RT-qPCR

cDNA synthesis was performed with one μg RNA using RNA to cDNA EcoDry Premix (Clontech). Primers used were as follows: Fig 1B PHF5A (cat# Hs00754435_s1, Invitrogen); Fig 2C luciferase (cat# Mr03987587_mr, Invitrogen); Fig 4B and 4C AAV polyA (Forward 5’-CCTGGGTTCAAGCGATTCTC-3’, Reverse 5’-AGCTGAGCCTGGTCATGCAT-3’, Probe 5’-/FAM/TGCCTCAGCCTCCCGAGTTGT, IDT).

### Western blotting

Western blotting was performed as described previously [64]. Following primary antibodies were used: rabbit anti-PHF5A (Sigma HPA028885-100UL) 1:50, rat anti-HA clone 3F10 (Roche 11867423001) 1:250, rabbit anti-VP1, 2, 3 (American Research Products, Inc. 03–61084) 1:250, mouse anti-SAP155 (SF3B1) (MBL International D221-3) 1:250. ImageJ software was used to quantify Western blots from immunoprecipitations.

### Southern blotting

DIG High Prime DNA Labeling and Detection Starter Kit II (Roche) was used for Southern blotting to detect the luciferase DNA in the AAV vector genome. A luciferase DNA fragment from pSIN-Luc was labeled according to the manufacturer’s instruction. HeLa cells were seeded in a 6-well plate at 200,000 cells per well, followed by transfection with control or PHF5A siRNAs. 24 hours post transfection, AAV9 CMV-Luc (MOI 8 x 10^4^) was added for 1, 3 or 6 hours. Cells were harvested in lysis buffer for nuclear fractionation. Nuclear lysates were purified as in “Cell fractionation and analysis of nuclear rAAV genomes” section. 2 μg of DNA sample was run on a 2% agarose gel without ethidium bromide at 50V for 1.5 hours. The gel was prepared for transfer in the following washes with rocking: 0.25N HCl 10 min, rinse ddH20, denaturation buffer (0.5N NaOH, 1.5M NaCl) 15 min, denaturation buffer 30 min, neutralization buffer (0.5M Tris, 1.5M NaCl) 15 min, and neutralization buffer 30 min. The gel was then blotted overnight by capillary transfer with 10x SSC on a positively charge nylon membrane (Roche). DNA was fixed to the membrane by UV-crosslinking and the luciferase probe was hybridized to the DNA overnight at 43.5°C. The membrane was washed and developed according to the protocol (Roche).

### Northern blotting

Cells were prepared for Northern blot by seeding at 200,000 cells per well in a 6-well plate, transfected with control and PHF5A siRNAs, followed by transduction with AAV9 CMV-Luc vector (MOI 4 x 10^5^). 36 hours post transduction cells were harvested and nuclear and cytoplasmic RNA was isolated using the PARIS kit (Ambion). 1 μg RNA was run on a formaldehyde gel, washed, and blotted by capillary transfer overnight according to the DIG Northern Starter Kit (Roche). The RNA was fixed to the membrane by UV-crosslinking and the DIG-labeled luciferase DNA probe from Southern blotting was incubated with the pretreated membrane overnight at 50°C. The membrane was washed and developed according to the manufacturer’s instructions.

### Cell fractionation and analysis of nuclear rAAV genomes

Cells were resuspended in cytoplasmic lysis buffer (1.3M sucrose, 20mM MgCl_2_, 4mM Tris, 4.2% Triton X-100) and incubated on ice for 10 min. The lysates were homogenized using a 21-guage needle and syringe, spun at 14,000 rpm for 15 min at 4°C and the supernatant (cytoplasmic fraction) was collected. The pellet (nuclear fraction) was washed with PBS. RNA was eliminated by RNaseA (200U/mL) treatment. A Qiagen QIAamp DNA Mini kit was used to further purify the DNA. Quantitative real-time PCR was performed to determine AAV luciferase genomic copy numbers. To assess encapsidated AAV genomes in the nucleus, the above procedure was followed with the addition of DNase (Invitrogen) treatment at 37°C for 30 min (both control and DNase treated samples) at the beginning of DNA purification and quantitative real-time PCR detection.

### Transgene expression from single-stranded AAV genomic DNA

AAV genomic DNA was isolated using the QIAamp DNA mini kit following the provided Protocols for Viral DNA (Qiagen). The genomic DNA was isolated from 4 x 10^11^ vector genomes of purified AAV9 CMV-Luc vector. HeLa cells transfected with control or PHF5A siRNAs were transfected with the purified AAV genomic DNA (0.1 μg/well) by FuGENE6 (Promega), and luciferase expression was analyzed 48 hours after viral DNA transfection.

### Immuno-precipitation

Semi-confluent HeLa cells with or without stable overexpression of the HA-tagged PHF5A were infected with AAV2 or AAV9 CMV-Luc (MOI 4 x 10^5^) in a 6-well plate for 6 hours at 37°C. Cells were then harvested on ice in RIPA buffer containing protease inhibitor, followed by pull-down with 20 μL anti-HA agarose beads (Pierce). After 15 cycles of washing, pellets were resuspended in 0.5 mL PBS and split into 2 aliquots. One aliquot was used for Western blotting of AAV capsid proteins, and the other was used for the isolation of total DNA by QIAamp DNA Mini kit for RT-qPCR detection of AAV genomic DNA. For pull-down assay using heated AAV particles, 100 μL cell lysate was combined with 3 x 10^10^ gc of AAV2 CMV-Luc vector particles that were unheated or pre-heated for 30 min at 65°C, followed by precipitation by anti-HA agarose as above.

### Confocal microscopy

The Lab-TekII 8-well chamber slides (Thermo Fisher Scientific) were pretreated for 5 min with poly-d-lysine (Sigma, 0.1 mg/mL). HeLa cells were plated at 1 x 10^4^ cells/well and were infected with AAV2 CMV-Luc (4 x 10^10^ gc/well) or empty AAV2 (25 μl/well) at 37°C for 5 min, 4 hours, or 12 hours. Cells were fixed in 4% paraformaldehyde for 20 min at room temperature, permeabilized with 0.3% Triton X-100 for 15 min, and blocked with 5% FBS/PBS for 30 min. Primary antibody was added, and cells were incubated for 1.5 hours at room temperature in a humidified chamber. Secondary antibody followed according to the same procedure. Then cells were washed three times with PBS, treated with DAPI (Sigma, 1:2000) for 1 min, washed three times with PBS, and mounted with Dako fluorescent mounting media. Confocal microscopy was performed on an LSM 780 confocal microscope (Zeiss). The following primary and secondary antibodies were used for immunocytochemistry of uninfected and AAV infected HeLa cells: anti-AAV particles (A20) mouse monoclonal antibody (American Research Products) at 1:100 followed by FITC-conjugated donkey anti mouse IgG (H+L) (Jackson 715-095-151) 1:500; rabbit anti-PHF5A (Sigma HPA028885-100UL) 1:250 and Alexa Fluor 594-conjugated donkey anti-rabbit IgG (H+L) (Invitrogen A-21207) 1:2000; rabbit anti-phospho-SF3B1 (MBL International PD043) 1:500 and Alexa Fluor 594-conjugated donkey anti-rabbit IgG (H+L) 1:500.

### Iodixanol cushion co-precipitation assay

AAV2 CMV-Luc vectors (5 x 10^10^ gc/tube) and purified AAV2 empty, VP3 only particles were left unheated or pre-heated at 65°C for 30 min and placed on ice. HeLa and HeLa-PHF5A-HA cells were harvested by incubating 1 well of a 6-well plate with 500 μL RIPA buffer supplemented with protease inhibitors on ice for 10 min. Cells were harvested by scraping, homogenized using a 21-guage needle and syringe, and spun for 5 min at 13,200 rpm. 400 μL of the cell lysate was added to the virus and samples were rotated for 1 hour at 4°C. The 25% iodixanol solution was prepared using 3.2 mL 1x PBS, 2.8 mL 9:1 Optiprep to 10x PBS, and 0.15% phenol red. 30 μL of the lysate virus mixture was removed and used as an input. The remaining lysate-virus mix was layered on top of 0.5 mL 25% iodixanol. These samples were spun at 4°C for 1 hour at 14,000 rpm. After spinning the clear upper layer, red lower layer, and pellet were harvested for Western blot. The following antibodies were used in this experiment: rabbit anti-PHF5A (Sigma HPA028885) 1:100, rat anti-HA clone 3F10 (Roche 11867423001) 1:500, rabbit anti-VP1, 2, 3 (American Research Products, Inc. 03–61084) 1:250, mouse anti-SF3B1 (MBL International D221-3) 1:500, rabbit anti-phospho-SF3B1 (MBL International PD043) 1:400, rabbit anti-histone H2B (Cell Signaling #8135) 1:1000, and rabbit anti-histone H3 (Cell Signaling #4499) 1:1000 in blocking buffer. To note, the membrane that received phospho-SF3B1 primary antibody was blocked in 5% BSA/PBS + 0.05% Tween-20. Antibodies were diluted in 1x TBS + 0.001% Tween-20.

### Verification of inhibition of pre-mRNA splicing by inhibitors

HeLa cells at 80% confluency were treated with pre-mRNA splicing inhibitors at the following concentrations: 3-Aminophenylboronic acid (5mM and 1mM), Isoginkgetin (25uM and 12.5uM), and meayamycin B (10nM and 5nM). Eight hours post drug RNA was isolated using TRIzol (Invitrogen), and cDNA synthesis was performed with one μg RNA using RNA to cDNA EcoDry Premix (Clontech). cDNA was amplified by KOD Hot Start DNA Polymerase (EMD Millipore) using primers for MAPT exon 10 5’-AAGATCGGCTCCACTGAGAA-3’ and 5’-ATGAGCCACACTTGGAGGTC-3’.

## Supporting Information

S1 Fig Characterization of RAB40A and PRICKL4 disruptions on AAV vector transduction. (A) Quantitative real-time RT-PCR was performed to determine the levels of RAB40A and PRICKLE4 transcripts in HeLa cells infected with a control lentiviral vector pLKO.1 or vectors carrying RAB40A and PRICKE4-targeting shRNAs at 48 hours. (B) Upon infection with AAV9 CMV-Luc vector for two days, relative luciferase activity was determined in HeLa cells pre-treated with the shRNA lentivectors. (C) Same as B, but luciferase-expressing adenoviral and lentiviral vectors were used. Data are shown as averages of three independent experiments with error bars representing standard error of the mean. * (p<0.05). For the introduction of shRNAs, cells were transduced with lentiviral control vector (pLKO.1), or vectors carrying shRAB40A or shPRICKLE4 from OpenBiosystems (plasmid names) at estimated MOI of 4. 24 hours post lentiviral transduction, cells were infected with luciferase-expressing vectors. Luciferase expression was measured by the ONE-Glo Luciferase Assay System (Promega) according to the manufacturer’s instruction. Pre-designed primers from Invitrogen were purchased (RAB40A, 4331182 Hs00369904_m1; PRICKLE4, 4331182 Hs00255728_m1) and used for the qRT-PCR. Over-expression of the PRICKLE4-Escape mutant did not reverse the effects of the PRICKLE4 shRNA in HeLa cells stably expressing an shRNA-resistant PRICKLE4 mutant. Thus, the effects observed with the PRICKLE4 disruption were likely due to off-target effects. We have not tested the effect of over-expression of RAB40A-Escape mutant.
(PDF)

S2 Fig Verification of the cytoplasmic and nuclear fractions by immunoblotting. Cytoplasmic and nuclear fractions were analyzed 2 hours p.i. to determine subcellular fractionation purity. GAPDH (cat# AM4300, Ambion, 1:1000) and Histone H3 (cat# 4499, Cell Signaling, 1:1000) antibodies were used as cytoplasmic and nuclear markers to verify the cytoplasmic and nuclear fractions respectively.
(PDF)

S3 Fig GATA-type zinc fingers in PHF5A are essential for the restriction of AAV vectors. (A) The alignment of PHD finger-like domains of PHF5A and its yeast homolog Rds3 is shown. Five conserved CxxC repeats are underlined. (B) Predicted triquetra knot structure of PHF5A, based on the yeast Rds3 structure (van Roon et al., PNAS 2008, 105:9621–6), is shown with the three GATA-type zinc fingers. (C) HeLa cells were transduced by lentiviral vectors expressing a series of zinc finger mutants of the PHF5A-HA-Escape, followed by puromycin selection. Expression of HA-tagged PHF5A proteins and their resistance to the PHF5A siRNA were verified by transfecting individual HeLa lines with control or PHF5A siRNAs. (D) Control HeLa cells, HeLa lines stably expressing PHF5A-HA Escape, or PHF5A-HA Escape zinc finger mutants, were pre-treated with control or PHF5A siRNAs for 24 hours, followed by transduction by the AAV9 CMV-Luc vector. Relative luciferase expression was determined 48 h ours p.i. Data are shown as averages of three independent experiments with error bars representing standard error of the mean.
(PDF)

S4 Fig Genetic and pharmacological inhibition of spliceosomal proteins on AAV vector transduction. (A) HeLa cells were transfected with control or siRNAs targeting the potential PHF5A-interacting proteins. Commercially available specific primers and probe sets were used for the quantitative real-time RT-PCR to determine the transcript levels. From Bio-Rad LMNA, FUS, EEF1A1, EEF2, HIST1H4B, U2AF1, SRSF5, SF3B1, SF3B2, SF3B3, EP400, DDX1, SNRNP200 and PRPF31 primers; from Invitrogen PHF5A primers and probe. The levels of individual transcripts in untreated cells were set as 100%. Error bars represent standard deviation. (B) HeLa cells were transfected with control siRNA, or a series of siRNAs for 24 hours, followed by the AAV9 CMV-Luc vector transduction. Relative luciferase expression was determined 48 h ours p.i. (C) HeLa cells were transduced with AAV2 or AAV9 CMV-Luc (MOI 10^4^) and given meayamycin (20 nM) 9 hours p.i. Luciferase assay was performed 48 hours p.i. (D) HeLa cells were transduced with AAV9 CMV-Luc vectors, in the presence of increasing doses of ABA. Relative luciferase expression was determined 72 hours p.i. (E) HeLa cells were transduction with AAV2 or AAV9 CMV-Luc vectors, followed by 20 nM meayamycin B treatments at various time points (3–24, 3–48, 3–72, 24–48, 24–72, or 48–72 hours p.i.). Cells were harvested and relative luciferase expression was determined 72 hours p.i. (F) Co-treatment of HeLa cells with PHF5A siRNAs and Meayamycin B. HeLa cells were treated with the siRNA for 48 hr, followed by infection with AAV9 CMV-Luc (MOI 10^4^). At 9 hours p.i. Meayamycin B (5nM) was added, and cells were harvested for the luciferase assay 48 hours p.i. (G) Influence of dual treatment with human adenovirus 5 infection and meayamicin on AAV vector infection. HeLa cells were infected with AAV2 CMV-Luc (MOI 10^4^) or co-infection with AAV2 CMV-Luc and human adenovirus 5 (MOI 3 x 10^4^), in the presence or absence of meayamycin (20 nM) for 48 hours. (H) Influence of adenovirus 5 infection on subcellular localization of PHF5A in HeLa cells. HeLa cells stably expressing the HA-tagged PHF5A were infected with human adenovirus 5 (MOI 10^4^) for 24 hours, and PHF5A-HA in control and infected HeLa cells was visualized by anti-HA antibody (red). (I) Influence of U2 snRNP inhibition on AAV vector production. AAV2 vectors were made by triple transfection with pHelper, pXX2, and pAAV CMV-Luc, triple transfection with Meayamycin B (5 nM), or double transfection (no pHelper) with Meayamycin B (5 nM). Viruses were added to HeLa cells (2 uL/well) and 48 hours later their infectivity was determined by luciferase assay.
(PDF)

S5 Fig Pull-down of the HA-tagged PHF5A protein. (A) HeLa or HeLa overexpressing PHF5A-HA lysates were used to pull-down the HA-tagged PHF5A by anti-HA agarose beads. After immunoprecipitation samples were spun for 5 min and the supernatant and pellet were separated and run on a Western blot for HA detection of the PHF5A-HA tagged protein. Relative PHF5A-HA levels were quantified using ImageJ software. (B) Same as A except samples were washed 15 times following IP and washes 5, 10 and 15 were saved an run along side the input and pull-down for Western blotting. The percent PHF5A-HA pulled down relative to the input was calculated by ImageJ software.
(PDF)

S6 Fig Subcellular localization of PHF5A. (A) Control and AAV2 vector-treated HeLa cells were monitored for the subcellular localization of endogenous PHF5A. Note increased cytoplasmic PHF5A signals upon AAV2 vector infection. (B) Same as Fig 4E, but without AAV vector infection. (C) Same as Fig 4E, but with (right) or without (left) pre-treatment with siRNA-PHF5A.
(PDF)

S7 Fig Influence of disruption of PHF5A expression on AAV vector trafficking in HeLa cells. (A) Control HeLa cells were infected with the AAV2 vector for 4 hours. AAV2 vector particles were detected by anti-AAV2 capsid A20 antibody, and the patterns of cytoplasmic and nuclear accumulations of AAV2 vector particles were visualized using Z-stack images. Top (slice 1) to bottom (slice 5) images were shown above. The middle sections (slices 3 and 4) were shown in higher magnification. (B) Same as A, but HeLa cells were pre-treated with the PHF5A siRNA for 24 hours. (C) Control and PHF5A siRNA-treated HeLa cells were infected with the AAV2 CMV-Luc vector for 4 hours, followed by confocal microscopic analysis of AAV2 capsid and PHF5A. Note the PHF5A siRNA-treatment suppressed the PHF5A signals. Again, no notable changes in AAV2 vector particle trafficking were observed.
(PDF)

S8 Fig Localization of SF3B1 and AAV2 capsid. (A) HeLa cells were infected with the AAV2 CMV-Luc vector for 5 min, 4 hr, or 12 hr. Confocal microscopy analysis was performed to detect the subcellular localizations of intact AAV vector particles (green) and SF3B1 (red) by specific antibodies. Nuclei were counterstained by DAPI (blue). (B) HeLa cells were infected with the AAV2 CMV-Luc vector for 4 hours, and cells were analyzed for co-localization of AAV2 capsid and endogenous SF3B1 signals. (C) Same as Fig 4H, except that heated or unheated AAV2 CMV-Luc vectors or heated or unheated empty AAV2 vectors were used. SF3B1 was more efficiently co-precipitated with pre-heated AAV2 particles, suggesting that heat-induced conformational changes in AAV2 capsids increase the interaction. Empty AAV2 capsids were able to enrich SF3B1 in the pellets, suggesting again there is no role for AAV genomic DNA in the interaction between SF3B1 and AAV2 capsid. (D) Same as Fig 4H, except that HeLa cells stably transduced with PHF5A-HA were used and PHF5A-HA was probed by anti-HA antibody.
(PDF)
