# Supplementary figures and images for: An siRNA Screen Identifies the U2 snRNP Spliceosome as a Host Restriction Factor for Recombinant Adeno-associated Viruses
Source: PLoS Pathog. 2015 Aug 5;11(8):e1005082. doi: 10.1371/journal.ppat.1005082 (PMC4526370; doi:10.1371/journal.ppat.1005082)

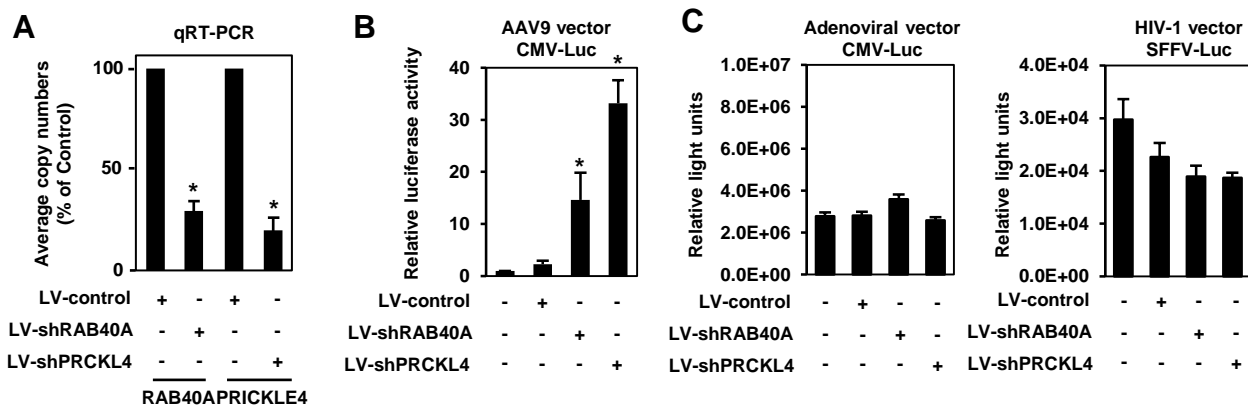

Supplement: S1 Fig — (A) Quantitative real-time RT-PCR was performed to determine the levels of RAB40A and PRICKLE4 transcripts in HeLa cells infected with a control lentiviral vector pLKO.1 or vectors carrying RAB40A and PRICKE4-targeting shRNAs at 48 hours. (B) Upon infection with AAV9 CMV-Luc vector for two days, relative luciferase activity was determined in HeLa cells pre-treated with the shRNA lentivectors. (C) Same as B, but luciferase-expressing adenoviral and lentiviral vectors were used. Data are shown as averages of three independent experiments with error bars representing standard error of the mean. * (p<0.05). For the introduction of shRNAs, cells were transduced with lentiviral control vector (pLKO.1), or vectors carrying shRAB40A or shPRICKLE4 from OpenBiosystems (plasmid names) at estimated MOI of 4. 24 hours post lentiviral transduction, cells were infected with luciferase-expressing vectors. Luciferase expression was measured by the ONE-Glo Luciferase Assay System (Promega) according to the manufacturer’s instruction. Pre-designed primers from Invitrogen were purchased (RAB40A, 4331182 Hs00369904_m1; PRICKLE4, 4331182 Hs00255728_m1) and used for the qRT-PCR. Over-expression of the PRICKLE4-Escape mutant did not reverse the effects of the PRICKLE4 shRNA in HeLa cells stably expressing an shRNA-resistant PRICKLE4 mutant. Thus, the effects observed with the PRICKLE4 disruption were likely due to off-target effects. We have not tested the effect of over-expression of RAB40A-Escape mutant. (PDF) [file ppat.1005082.s001.pdf]

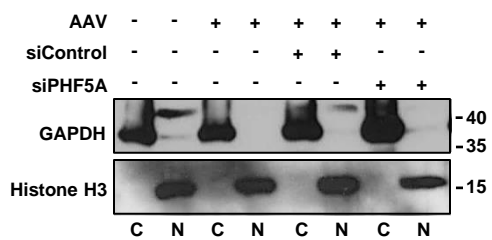

Supplement: S2 Fig — Cytoplasmic and nuclear fractions were analyzed 2 hours p.i. to determine subcellular fractionation purity. GAPDH (cat# AM4300, Ambion, 1:1000) and Histone H3 (cat# 4499, Cell Signaling, 1:1000) antibodies were used as cytoplasmic and nuclear markers to verify the cytoplasmic and nuclear fractions respectively. (PDF) [file ppat.1005082.s002.pdf]

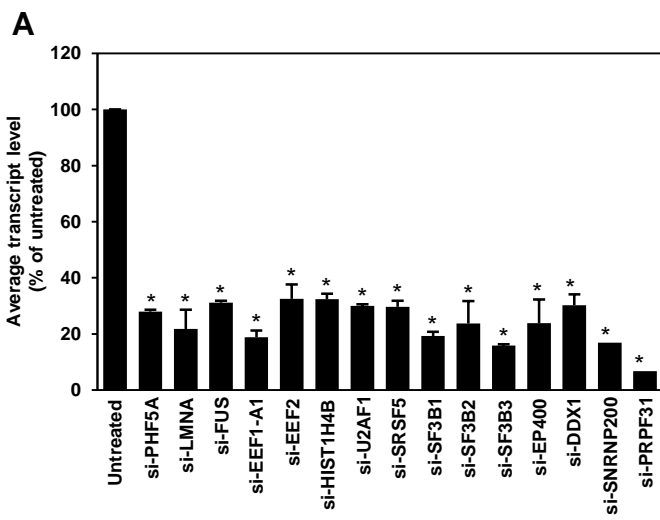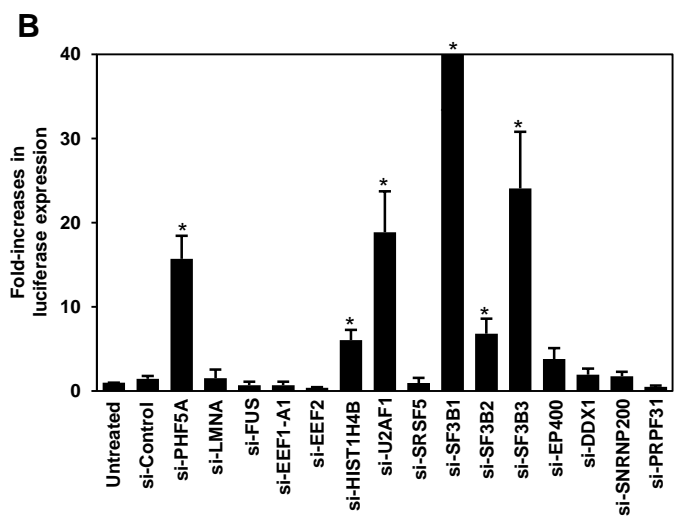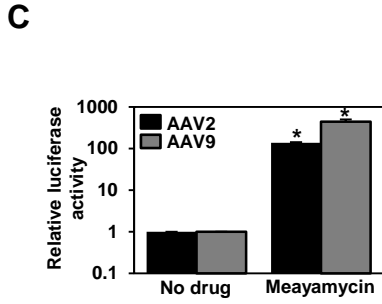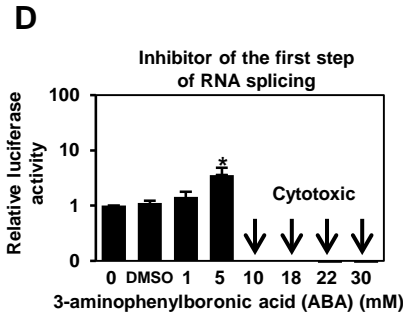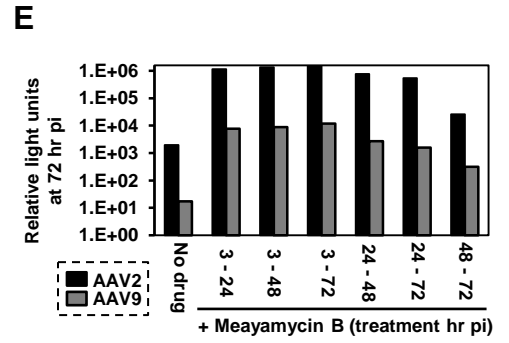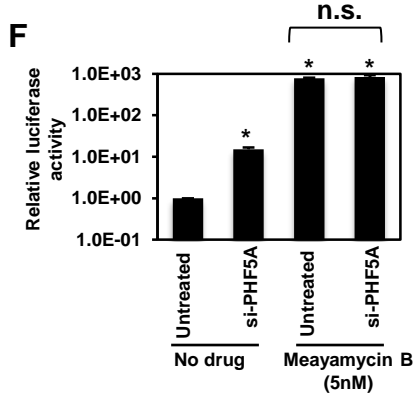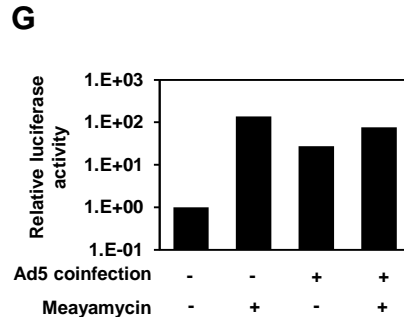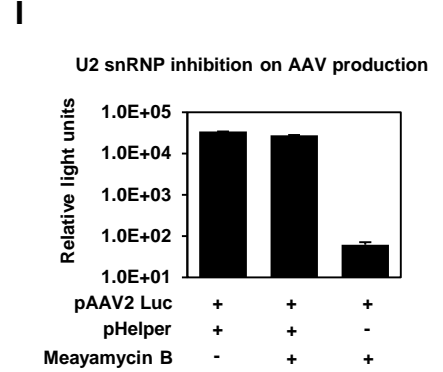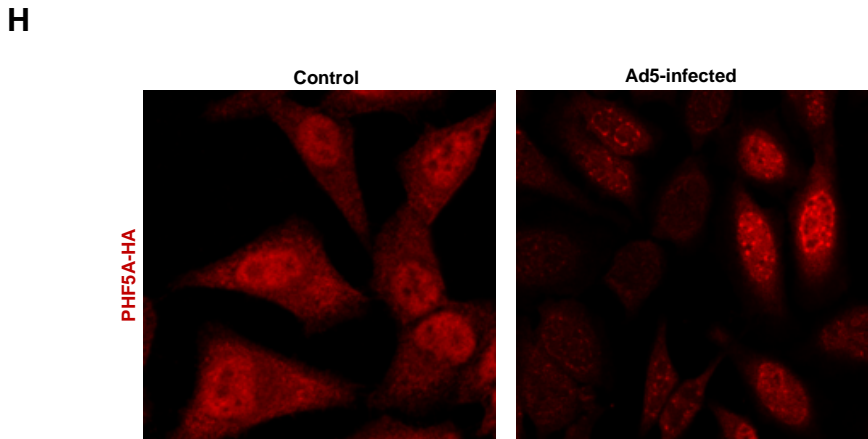

Supplement: S4 Fig — (A) HeLa cells were transfected with control or siRNAs targeting the potential PHF5A-interacting proteins. Commercially available specific primers and probe sets were used for the quantitative real-time RT-PCR to determine the transcript levels. From Bio-Rad LMNA, FUS, EEF1A1, EEF2, HIST1H4B, U2AF1, SRSF5, SF3B1, SF3B2, SF3B3, EP400, DDX1, SNRNP200 and PRPF31 primers; from Invitrogen PHF5A primers and probe. The levels of individual transcripts in untreated cells were set as 100%. Error bars represent standard deviation. (B) HeLa cells were transfected with control siRNA, or a series of siRNAs for 24 hours, followed by the AAV9 CMV-Luc vector transduction. Relative luciferase expression was determined 48 h ours p.i. (C) HeLa cells were transduced with AAV2 or AAV9 CMV-Luc (MOI 104) and given meayamycin (20 nM) 9 hours p.i. Luciferase assay was performed 48 hours p.i. (D) HeLa cells were transduced with AAV9 CMV-Luc vectors, in the presence of increasing doses of ABA. Relative luciferase expression was determined 72 hours p.i. (E) HeLa cells were transduction with AAV2 or AAV9 CMV-Luc vectors, followed by 20 nM meayamycin B treatments at various time points (3–24, 3–48, 3–72, 24–48, 24–72, or 48–72 hours p.i.). Cells were harvested and relative luciferase expression was determined 72 hours p.i. (F) Co-treatment of HeLa cells with PHF5A siRNAs and Meayamycin B. HeLa cells were treated with the siRNA for 48 hr, followed by infection with AAV9 CMV-Luc (MOI 104). At 9 hours p.i. Meayamycin B (5nM) was added, and cells were harvested for the luciferase assay 48 hours p.i. (G) Influence of dual treatment with human adenovirus 5 infection and meayamicin on AAV vector infection. HeLa cells were infected with AAV2 CMV-Luc (MOI 104) or co-infection with AAV2 CMV-Luc and human adenovirus 5 (MOI 3 x 10^4^), in the presence or absence of meayamycin (20 nM) for 48 hours. (H) Influence of adenovirus 5 infection on subcellular localization of PHF5A in HeLa cells. HeLa cells stably exp [file ppat.1005082.s004.pdf]

**A**

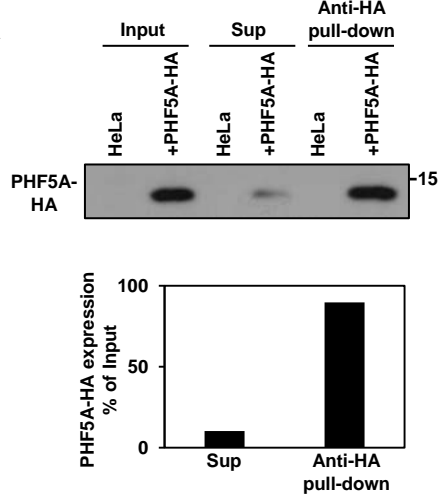

**B**

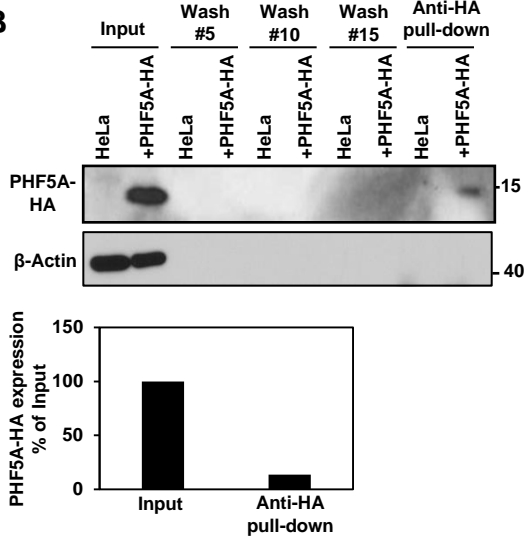

Supplement: S5 Fig — (A) HeLa or HeLa overexpressing PHF5A-HA lysates were used to pull-down the HA-tagged PHF5A by anti-HA agarose beads. After immunoprecipitation samples were spun for 5 min and the supernatant and pellet were separated and run on a Western blot for HA detection of the PHF5A-HA tagged protein. Relative PHF5A-HA levels were quantified using ImageJ software. (B) Same as A except samples were washed 15 times following IP and washes 5, 10 and 15 were saved an run along side the input and pull-down for Western blotting. The percent PHF5A-HA pulled down relative to the input was calculated by ImageJ software. (PDF) [file ppat.1005082.s005.pdf]

**A**

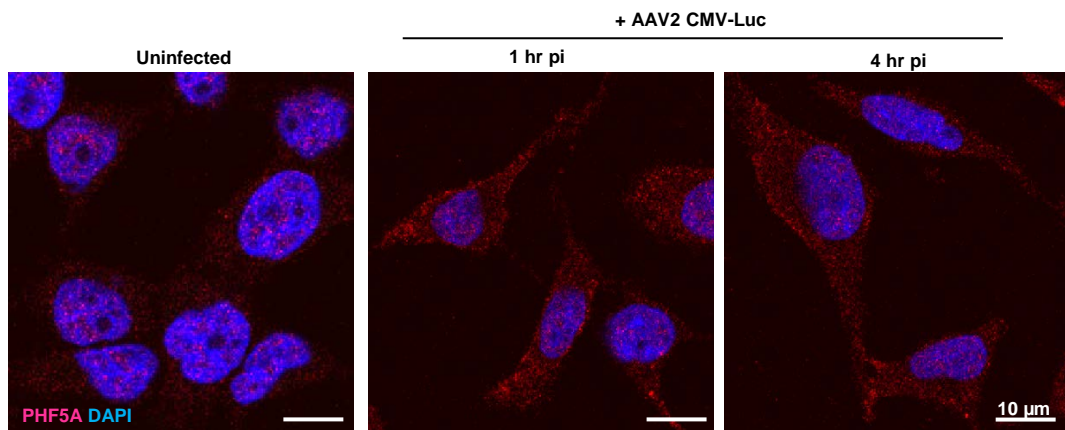

**B**

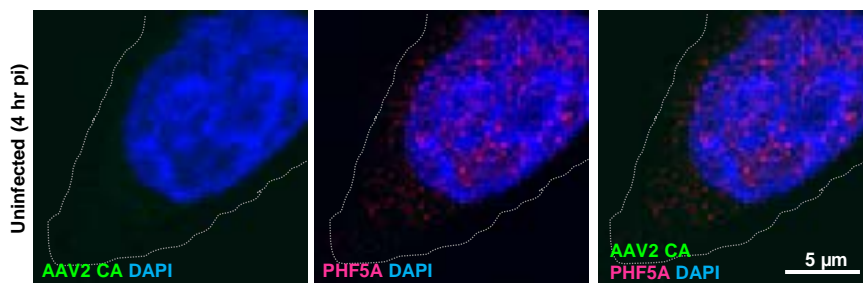

**C**

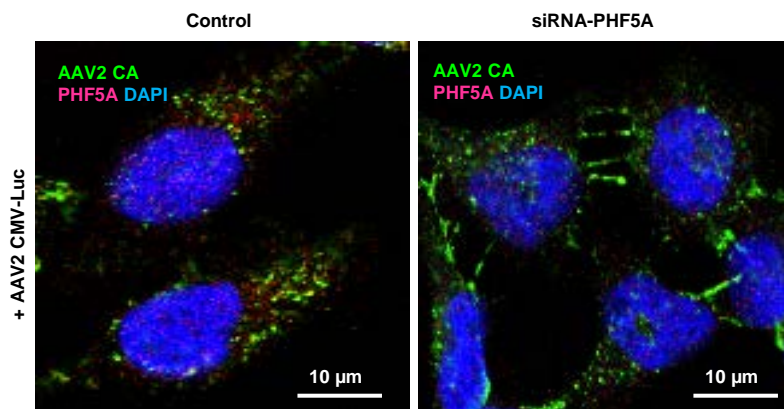

Supplement: S6 Fig — (A) Control and AAV2 vector-treated HeLa cells were monitored for the subcellular localization of endogenous PHF5A. Note increased cytoplasmic PHF5A signals upon AAV2 vector infection. (B) Same as Fig 4E, but without AAV vector infection. (C) Same as Fig 4E, but with (right) or without (left) pre-treatment with siRNA-PHF5A. (PDF) [file ppat.1005082.s006.pdf]

**A**

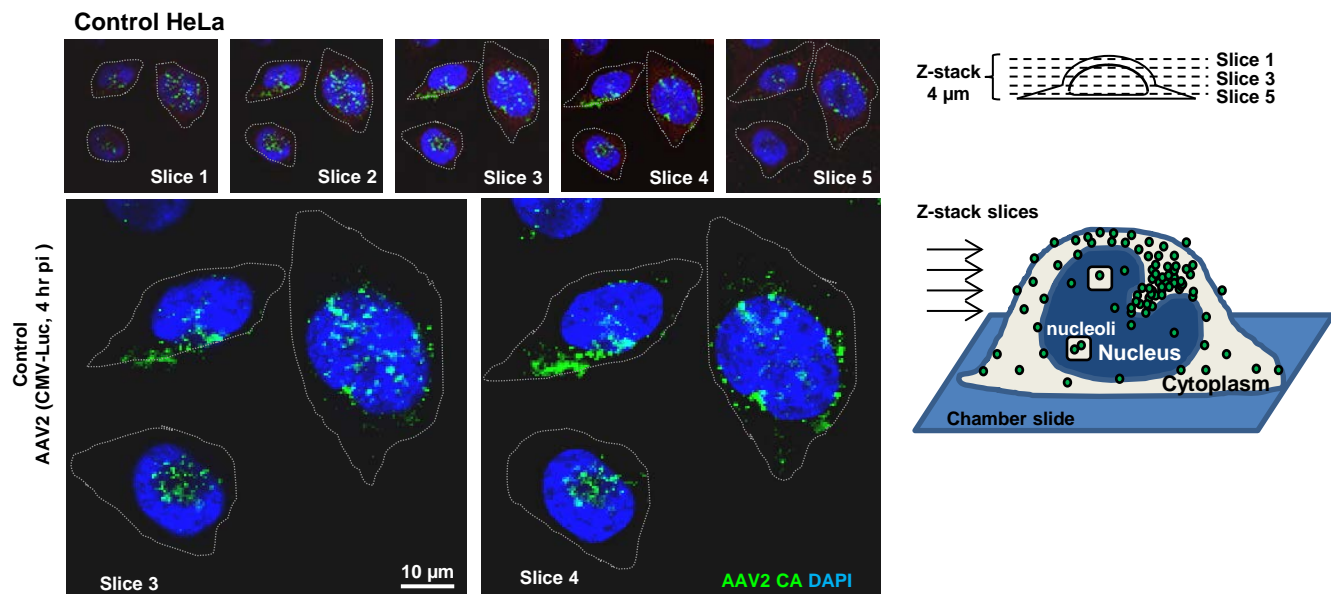

**B**

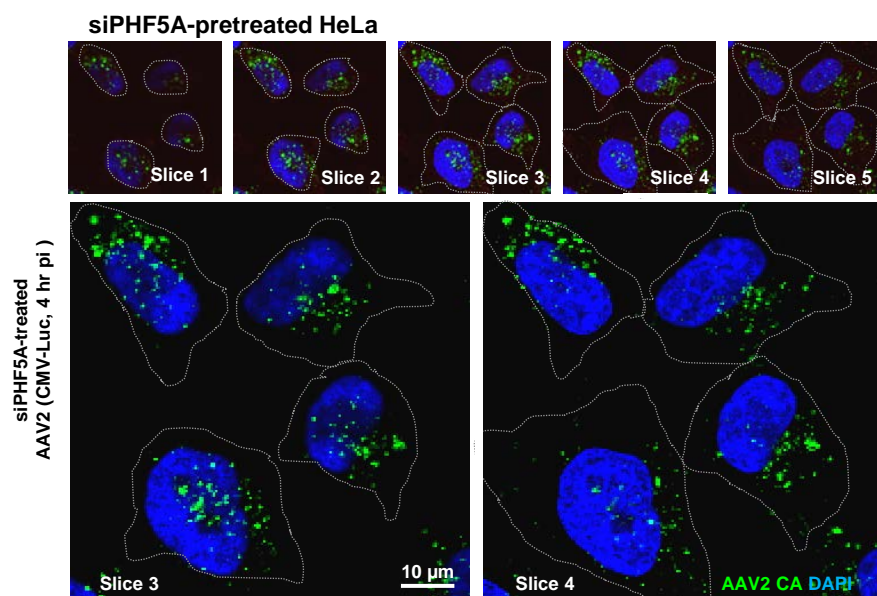

**C**

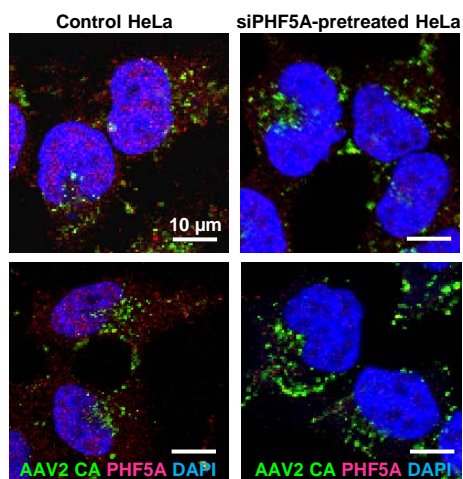

Supplement: S7 Fig — (A) Control HeLa cells were infected with the AAV2 vector for 4 hours. AAV2 vector particles were detected by anti-AAV2 capsid A20 antibody, and the patterns of cytoplasmic and nuclear accumulations of AAV2 vector particles were visualized using Z-stack images. Top (slice 1) to bottom (slice 5) images were shown above. The middle sections (slices 3 and 4) were shown in higher magnification. (B) Same as A, but HeLa cells were pre-treated with the PHF5A siRNA for 24 hours. (C) Control and PHF5A siRNA-treated HeLa cells were infected with the AAV2 CMV-Luc vector for 4 hours, followed by confocal microscopic analysis of AAV2 capsid and PHF5A. Note the PHF5A siRNA-treatment suppressed the PHF5A signals. Again, no notable changes in AAV2 vector particle trafficking were observed. (PDF) [file ppat.1005082.s007.pdf]

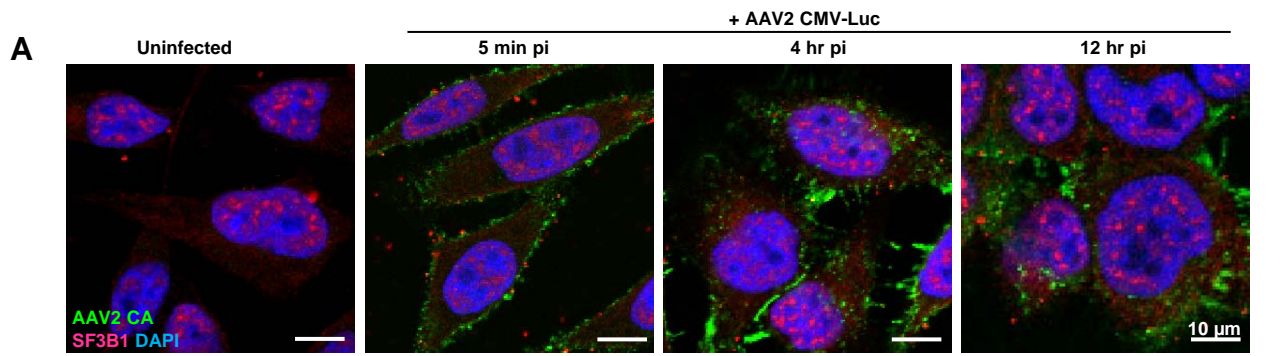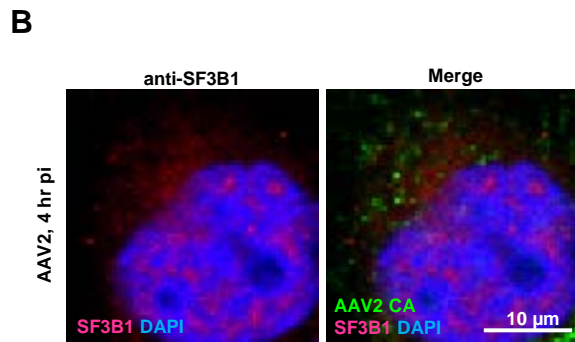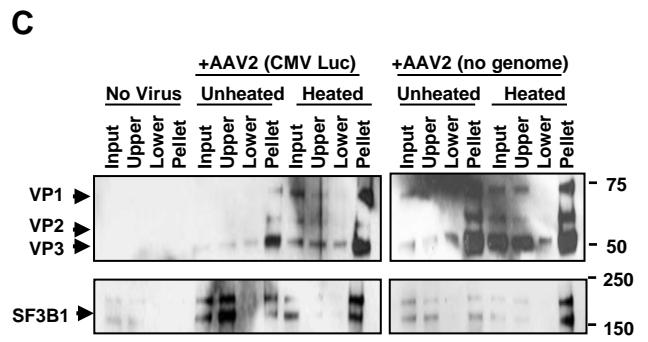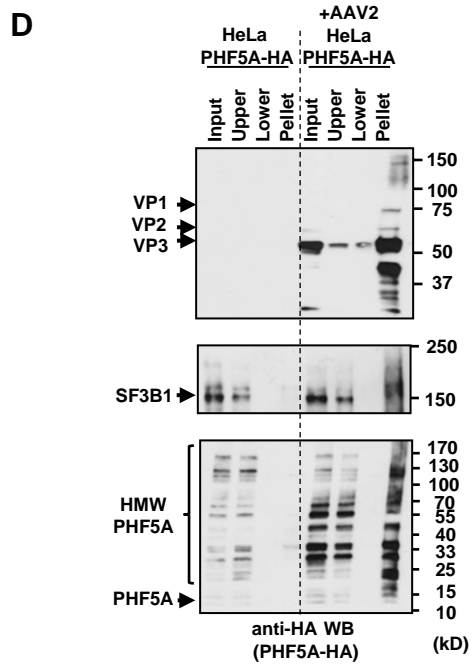

Supplement: S8 Fig — (A) HeLa cells were infected with the AAV2 CMV-Luc vector for 5 min, 4 hr, or 12 hr. Confocal microscopy analysis was performed to detect the subcellular localizations of intact AAV vector particles (green) and SF3B1 (red) by specific antibodies. Nuclei were counterstained by DAPI (blue). (B) HeLa cells were infected with the AAV2 CMV-Luc vector for 4 hours, and cells were analyzed for co-localization of AAV2 capsid and endogenous SF3B1 signals. (C) Same as Fig 4H, except that heated or unheated AAV2 CMV-Luc vectors or heated or unheated empty AAV2 vectors were used. SF3B1 was more efficiently co-precipitated with pre-heated AAV2 particles, suggesting that heat-induced conformational changes in AAV2 capsids increase the interaction. Empty AAV2 capsids were able to enrich SF3B1 in the pellets, suggesting again there is no role for AAV genomic DNA in the interaction between SF3B1 and AAV2 capsid. (D) Same as Fig 4H, except that HeLa cells stably transduced with PHF5A-HA were used and PHF5A-HA was probed by anti-HA antibody. (PDF) [file ppat.1005082.s008.pdf]
