# Supplementary material for: An siRNA Screen Identifies the U2 snRNP Spliceosome as a Host Restriction Factor for Recombinant Adeno-associated Viruses
Source: PLoS Pathog. 2015 Aug 5;11(8):e1005082. doi: 10.1371/journal.ppat.1005082 (PMC4526370; doi:10.1371/journal.ppat.1005082)
Supplement: S3 Fig — (A) The alignment of PHD finger-like domains of PHF5A and its yeast homolog Rds3 is shown. Five conserved CxxC repeats are underlined. (B) Predicted triquetra knot structure of PHF5A, based on the yeast Rds3 structure (van Roon et al., PNAS 2008, 105:9621–6), is shown with the three GATA-type zinc fingers. (C) HeLa cells were transduced by lentiviral vectors expressing a series of zinc finger mutants of the PHF5A-HA-Escape, followed by puromycin selection. Expression of HA-tagged PHF5A proteins and their resistance to the PHF5A siRNA were verified by transfecting individual HeLa lines with control or PHF5A siRNAs. (D) Control HeLa cells, HeLa lines stably expressing PHF5A-HA Escape, or PHF5A-HA Escape zinc finger mutants, were pre-treated with control or PHF5A siRNAs for 24 hours, followed by transduction by the AAV9 CMV-Luc vector. Relative luciferase expression was determined 48 h ours p.i. Data are shown as averages of three independent experiments with error bars representing standard error of the mean. (PDF) [file ppat.1005082.s003.pdf]

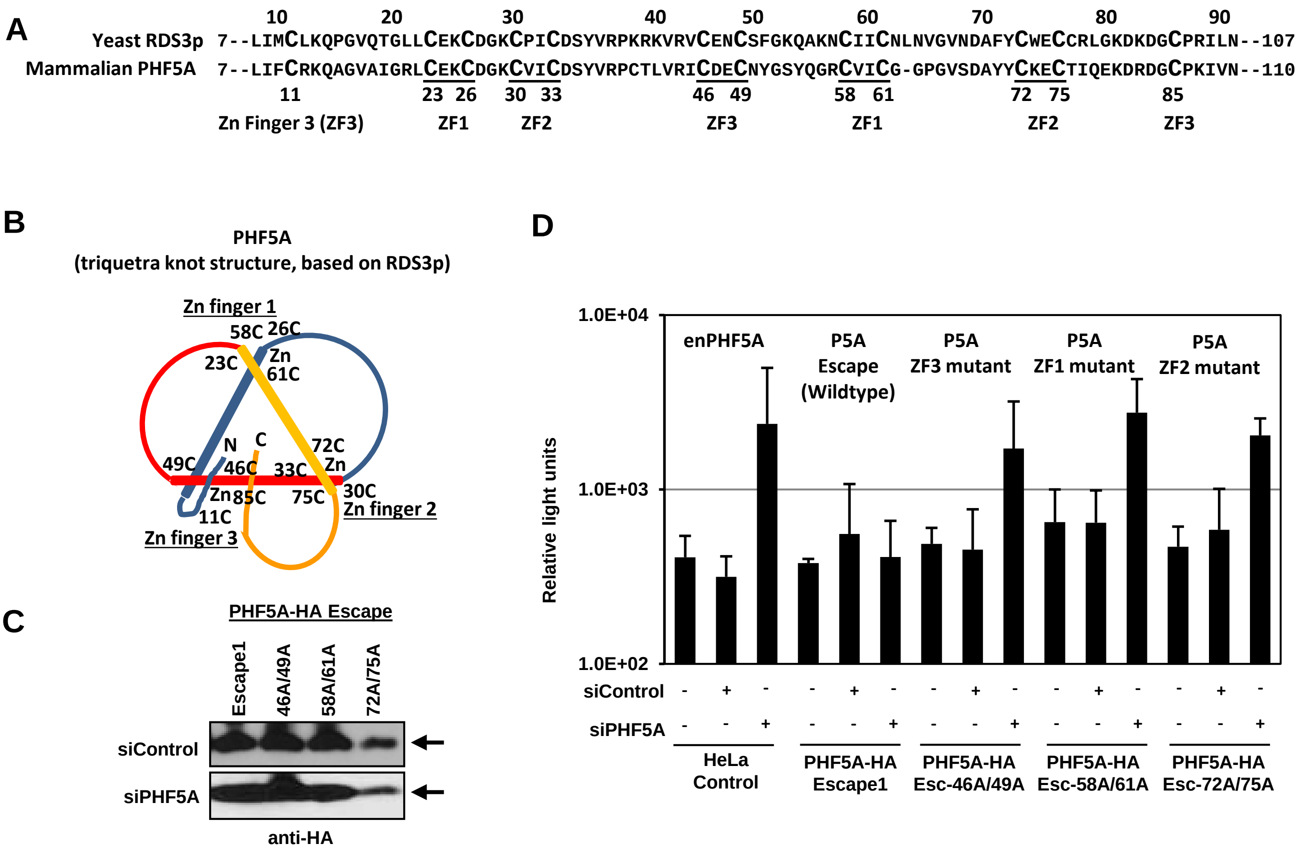
A
10 20 30 40 50 60 70 80 90
Yeast RDS3p 7--LIMCLKQPGVQTGLLCEKCDGKCPICDSYVRPKRKVRVCENCSFGKQAKNCIICNLNVGVNDAFYCWECCRLGKDKDGCPRILN--107
Mammalian PHF5A 7--LIFCRKQAGVAIGRLCEKCDGKCVICDSYVRPCTLVRICDECNYGSYQGRCVICG-GPGVSDAYYCKECTIQEKDRDGCPKIVN--110
11 23 26 30 33 46 49 58 61 72 75 85
Zn Finger 3 (ZF3) ZF1 ZF2 ZF3 ZF1 ZF2 ZF3
B
PHF5A
(triquetra knot structure, based on RDS3p)
Zn finger 1
58C 26C
23C
Zn
61C
N
C
72C
49C
46C
33C
Zn
Zn
85C
75C
30C
Zn finger 2
11C
Zn finger 3
C
PHF5A-HA Escape
Escape1
46A/49A
58A/61A
72A/75A
siControl
siPHF5A
anti-HA
D
Relative light units
1.0E+04
1.0E+03
1.0E+02
enPHF5A
P5A Escape (Wildtype)
P5A ZF3 mutant
P5A ZF1 mutant
P5A ZF2 mutant
siControl - + - - + - - + - - + - - + -
siPHF5A - - + - - + - - + - - + - - +
HeLa Control
PHF5A-HA Escape1
PHF5A-HA Esc-46A/49A
PHF5A-HA Esc-58A/61A
PHF5A-HA Esc-72A/75A
